# Sulfoquinovose is a select nutrient of prominent bacteria and a source of hydrogen sulfide in the human gut

**DOI:** 10.1038/s41396-021-00968-0

**Published:** 2021-03-31

**Authors:** Buck T. Hanson, K. Dimitri Kits, Jessica Löffler, Anna G. Burrichter, Alexander Fiedler, Karin Denger, Benjamin Frommeyer, Craig W. Herbold, Thomas Rattei, Nicolai Karcher, Nicola Segata, David Schleheck, Alexander Loy

**Affiliations:** 1 Division of Microbial Ecology, Centre for Microbiology and Environmental Systems Science, University of Vienna, Vienna, Austria; 2 FFoQSI GmbH, Austrian Competence Centre for Feed and Food Quality Safety & Innovation, Tulln, Austria; 3 Department of Biology, University of Konstanz, Konstanz, Germany; 4 Konstanz Research School Chemical Biology, University of Konstanz, Konstanz, Germany; 5 Division of Computational Systems Biology, Centre for Microbiology and Environmental Systems Science, University of Vienna, Vienna, Austria; 6 CIBIO Department, University of Trento, Trento, Italy; 7 Joint Microbiome Facility of the Medical University of Vienna and the University of Vienna, Vienna, Austria

**Keywords:** Microbiome, Bacterial physiology

## Abstract

Responses of the microbiota to diet are highly personalized but mechanistically not well understood because many metabolic capabilities and interactions of human gut microorganisms are unknown. Here we show that sulfoquinovose (SQ), a sulfonated monosaccharide omnipresent in green vegetables, is a selective yet relevant substrate for few but ubiquitous bacteria in the human gut. In human feces and in defined co-culture, *Eubacterium rectale* and *Bilophila wadsworthia* used recently identified pathways to cooperatively catabolize SQ with 2,3-dihydroxypropane-1-sulfonate as a transient intermediate to hydrogen sulfide (H_2_S), a key intestinal metabolite with disparate effects on host health. SQ-degradation capability is encoded in almost half of *E. rectale* genomes but otherwise sparsely distributed among microbial species in the human intestine. However, re-analysis of fecal metatranscriptome datasets of four human cohorts showed that SQ degradation (mostly from *E. rectale* and *Faecalibacterium prausnitzii*) and H_2_S production (mostly from *B. wadsworthia*) pathways were expressed abundantly across various health states, demonstrating that these microbial functions are core attributes of the human gut. The discovery of green-diet-derived SQ as an exclusive microbial nutrient and an additional source of H_2_S in the human gut highlights the role of individual dietary compounds and organosulfur metabolism on microbial activity and has implications for precision editing of the gut microbiota by dietary and prebiotic interventions.

## Introduction

Dietary habits largely modulate the highly personalized composition and temporal dynamics of the human intestinal microbiota and influence disease risk [1–3]. Each individual daily ingests thousands of different dietary compounds [4] of which some, such as starch or glucose, are substrates for many microorganisms while others, such as the seaweed polysaccharide porphyran, are select energy sources of metabolically specialized microorganisms [5, 6]. Disentangling the effects of single dietary compounds on gut microbiota membership and activity is important for understanding diet–microbiota interaction mechanisms [7] but complicated because the genetic and physiological capabilities of microorganisms are insufficiently established [8].

Here, we explored for the first time microbial metabolism of sulfoquinovose (6-deoxy-6-sulfoglucose, SQ) in the human gut. SQ is the polar head group of sulfoquinovosyl diacylglycerol (SQDG), a ubiquitous sulfolipid in the photosynthetic membranes of all land plants, algae, dinoflagellates, and cyanobacteria. SQDG is one of the most abundant organic sulfur compounds in the biosphere and can represent greater than 25% of the total lipids in common dietary, leafy green vegetables such as spinach, lettuce, and green onion [9, 10]. Studies on the catabolism of dietary SQDG by animals are scarce; in guinea pigs SQDG is deacylated via host-derived lipases to sulfoquinovosyl glycerol (SQG) [11]. It is not known whether host tissues are able to metabolize SQG or SQ. Some *Proteobacteria* can catabolize SQDG/SQG and SQ analogously to the Embden–Meyerhof–Parnas (EMP) and the Entner–Doudoroff (ED) glycolytic pathways, hence, either via the sulfo-EMP (e.g., commensal and pathogenic *Escherichia coli*) or the sulfo-ED pathway (e.g., *Pseudomonas putida*) [12–14]. Recently, a third pathway for SQ degradation via a novel 6-deoxy-6-sulfofructose transaldolase (SFT) was described in the aerobic *Bacillus aryabhattai* isolate SOS1 [15] and *Bacillus megaterium* DSM 1804 [16]. The intestinal strains of the Firmicutes species *Enterococcus gilvus*, *Clostridium symbiosum*, and *Eubacterium rectale* also expressed SFT pathway genes during anaerobic, fermentative growth with SQ [15]. Bacterial SQ-degradation products are the excreted C_3_-organosulfonates 2,3-dihydroxypropane-1-sulfonate (DHPS) or 3-sulfolactate, which can serve as sources of sulfite via diverse catabolic pathways of specialized sulfite-respiring and H_2_S-producing *Desulfovibrionaceae* species [17, 18]. Although mammalian cells also produce H_2_S in the cytoplasm and in mitochondria [19], bacteria that anaerobically respire sulfite derived from sulfate or taurine are considered the main sources of H_2_S in the intestine [20, 21]. Because SQDG is a frequent compound in a vegetarian diet and some gut bacteria have the catabolic potential for complete degradation of SQ to H_2_S via interspecies cross-feeding of 3-sulfolactate or DHPS, SQ could be a so far overlooked green-diet-derived source of H_2_S in the gut. H_2_S is a Janus-faced metabolite that exerts its manifold beneficial and harmful impacts on the host in a dose-dependent manner [22]. For example, H_2_S is a mitochondrial energy source, a cellular antioxidant, and an important signaling molecule in mammalian physiology [23]. In contrast, at higher concentrations, H_2_S can chemically deteriorate the mucosal barrier of the gastrointestinal tract [24] and decrease ATP production through inhibition of cytochrome *c*, potentially leading to cell death [25]. Diets high in saturated fat trigger increased excretion of taurocholic bile acids and promote intestinal expansion of the taurine-utilizing pathobiont *Bilophila wadsworthia*, exacerbating intestinal inflammation in a susceptible host [26]. Clearly, a more complete knowledge of the sources and sinks of H_2_S is required to understand what regulates its homeostasis in the gut and whether it is beneficial or detrimental to the host [20, 27].

In this study, we hypothesized that SQ is selectively degraded by specialized bacteria and contributes to the formation of H_2_S in the human gut. By targeted physiological experiments with human fecal microcosms and mono- and co-cultures and by re-analysis of large metagenome and metatranscriptome datasets from human fecal samples, we reveal the identity of bacteria involved in complete degradation of SQ to H_2_S, the activity of the underlying pathways, and provide evidence for their importance for the microbial processes in the gut.

## Materials and methods

Supplementary Materials and Methods provide further details on the methods described below.

### Chemicals

SQ and 3-sulfolactate were synthesized by MCAT GmbH (Donaueschingen, Germany). DHPS was synthesized and validated by NMR and HPLC–MS as reported previously [12]. Taurine, isethionate and all other purchased, routine chemicals were of at least *puriss* p.a. grade if not otherwise stated.

### Human fecal microcosms

Anaerobic microcosms containing human vegetarian fecal samples were used to evaluate SQ metabolism by the gut microorganisms. Microcosms were subsampled over 10 days for metabolite quantification and community composition analyses.

### Pure culture and defined co-culture experiments

*E. rectale* DSM 17629 and *B. wadsworthia* strain 3.1.6 were grown on SQ and DHPS, respectively, as described previously [15, 17, 28]. At intervals, cultures were subsampled for metabolite quantification. *B. wadsworthia* 3.1.6 cells were harvested from the late exponential growth phase for differential proteomics when grown with the four different organosulfonates or for preparation of anoxic cell-free extracts for DHPS sulfite-lyase enzyme tests. For co-cultivation experiments, *E. rectale* DSM 17629 was inoculated first (*n* = 3) and after 81 h *B. wadsworthia* 3.1.6 was co-inoculated.

### Anoxic cell-free extracts and enzyme tests

DHPS sulfite-lyase assays using cell-free *B. wadsworthia* 3.1.6 extracts were done as described previously [28] with colorimetric quantification of sulfite and HPLC-UV analysis of derivatizatized hydroxyacetone.

### Differential proteomics

Proteomic analyses of cell-free *B. wadsworthia* 3.1.6 extracts were done as previously described [28].

### Metabolite analyses

H_2_S was quantified colorimetrically [29]. Short-chain fatty acids were quantified using capillary electrophoresis. SQ, DHPS, 3-sulfolactate, taurine, and isethionate were quantified as described previously [12]. Hydroxyacetone, short-chain fatty acids, and alcohols in culture experiments were analyzed by an HPLC method described previously [17]. Sulfite in enzyme reactions was quantified using a colorimetric assay (Fuchsin assay) or by derivatization with *N*-(9-acridinyl)maleimide and HPLC-UV [28].

### Fluorescence cell counting

FISH probes targeting *E. rectale* and *B. wadsworthia* were designed using ARB [30] with optimal hybridization conditions determined on pure cultures of *E. rectale* DSM 17629 and *B. wadsworthia* DSM 11045 (Table S1). Aliquots of PFA-fixed microcosm biomass were filtered onto black polycarbonate filters, stained with FISH probes, and counted using standard procedures [31].

### Single-cell stable-isotope probing by FISH-Raman microspectroscopy

PFA-fixed cells from microcosms incubated with 10 mM SQ and 50% D_2_O were subjected to liquid-based FISH [32]. Samples were spotted onto an aluminum-coated glass slide and after air drying fluorescence microscopy was used to identify cells for Raman analyses. Single-cell Raman spectra were acquired using a LabRAM HR800 confocal Raman microscope (Horiba Jobin-Yvon) and D-labeling (%CD) was quantified from integrated C-D and C-H peak areas as previously described [32].

### Extraction of nucleic acids

DNA for amplicon and metagenome sequencing and RNA for metatranscriptome sequencing were extracted following standard procedures [33] that included bead beating for 30 s on dry ice using a Lysing Matrix E tube (MP Biomedicals). Purified nucleic acids were stored in 30 µl DNase/RNase-free H_2_O at −80 °C.

### 16S rRNA gene and *dsrB* amplicon sequencing

Established two-step PCR barcoding protocols were used for amplicon sequencing of the 16S rRNA gene [34] and *dsrB* [35]. Barcoded libraries were pooled at equivalent copy numbers (20 × 10^9^) for 300 bp paired-end sequencing on a MiSeq sequencer (Illumina). Sequencing results were analyzed according to the procedures outlined previously [34, 35].

### Metagenomics

Samples from three time points (28, 73, and 114 h) from SQ-amended microcosms were chosen for 150 bp paired-end metagenome HiSeq 3000/4000 (Illumina) sequencing. Trimmed and error-corrected reads were assembled using metaSPAdes [36], and metagenome-assembled genomes (MAGs) were binned using MetaBAT [37]. MAGs were assessed for quality using checkM [38], de-replicated using drep [39], and assigned taxonomy through alignment and phylogenetic placement of concatenated marker genes into the Genome Taxonomy Database reference tree (GTDB-Tk) [40]. Average nucleotide identity comparisons between genomes were calculated using FastANI [41].

### Metatranscriptomics

Metatranscriptome libraries were prepared from three SQ-amended microcosm time points (6, 20, and 52 h). Following DNase digestion, rRNA was removed and 1–100 ng was used in each library preparation for multiplexed Illumina RNASeq analysis. Sequencing (50 bp, single-end) was performed using HiSeq 3000/4000 (Illumina). Trimmed reads were mapped to the genomes of *E. rectale* ATCC 33656 and *B. wadsworthia* 3.1.6, because these genomes are complete and have a very high ANI to the respective *E. rectale* and *B. wadsworthia* MAGs obtained from the microcosms. Feature counting and differential expression analysis was done using featureCounts within the Subread package [42] and the exactTest function (edgeR), respectively.

### Construction of a bacterial YihQ/SftG profile hidden markov-model (HMM)

Bacterial genomes and MAGs in Genbank as of July 27, 2017 (*N* = 103411) were screened using hmmsearch [43] for genes that contained the glycoside hydrolase (GH) family 31 pfam (PF01055.25). The resulting dataset (*N* = 127,608) was screened using a YihQ/SftG-specific motif that we constructed using signature residues reported by Speciale et al. [44]. A manually curated alignment was used to construct an HMM using hmmbuild [43] (File S1).

### Genome analyses and comparative genomics

All contigs and MAGs from the SQ incubations were screened for known SQ metabolism genes using blastp and the YihQ/SftG HMM. In addition, we searched the NCBI genome database using blastp for organisms that contain the four essential pathway genes—the sulfoquinovosidase (YihQ/SftG), SQ isomerase (SftI), 6-deoxy-6-sulfofructose transaldolase (SftT), and the reductase (SftR) (Table S2). The presence of the SFT gene cluster in 73,798 high-quality MAGs from publicly available human gut metagenomes [45] was searched using a two-step approach (Table S3). First, translated open reading frames were queried with the YihQ/SftG HMM. Second, blast-based searches for SftI, SftT, and SftR were performed against all translated open reading frames of YihQ/SftG-containing genomes. Genes enabling taurine (*tpa*), DHPS (*hpsGH, hpsO, hpsN, dphA*), 3-sulfolactaldehyde (*slaB*), 3-sulfolactate (*suyAB, slsC, comC*), sulfoacetaldehyde (*xsc, sarD*), and isethionate (*islAB*) catabolism and sulfite reduction (*dsrABC*) were identified in 16 representative *dsrAB*-containing human gut bacteria by blastp screening.

### Metatranscriptome analysis of publicly available datasets

Reads from 1,090 paired gut metagenomes/metatranscriptomes were trimmed followed by taxonomic and functional profiling using HUMAnN2 [46] and MetaPhlAn2 [47]. Contributions of SFT pathway-encoding organisms to SFT pathway expression were assessed by mapping of the trimmed reads from the 1,090 metatranscriptomes to the SFT pathway-encoding genomes identified in our NCBI search. A similar approach was used to assess expression of *hpsGH*, *islAB*, and *dsrABC* genes in the 16 representative *dsrAB*-containing human gut bacteria (see previous section). The relative abundance of the SFT pathway in the 1,090 metatranscriptomes was evaluated using HUMANn2. Proportional contributions of each organism to total expression of the SFT pathway was calculated as a ratio between the RPKM value for the organism of interest divided by the total RPKM value for the pathway in that sample.

### *E. rectale* transcription network analysis

Trimmed reads (processed as described above) from the 1090 gut metatranscriptomes were mapped to the *E. rectale* ATCC 33656 genome. Samples with greater than 5/8 of all *E. rectale* ATCC 33656 genes with at least 1 read mapped (*n* = 244) were selected for network construction. A read count-normalized matrix was then used as input into SPIEC-EASI [48]. Significant correlations between gene pairs (positive or negative interaction strength of >0.099) with any functional prediction (*n* = 1349 unique genes, 2326 gene pairs) were then imported into Cytoscape [49] for network visualization. Significant clusters were identified using ClusterONE [50].

### Phylogenetic and phylogenomic tree construction

Phylogenomic analysis of the 93 MAGs from the fecal incubations was performed with GToTree [51] using 74 bacterial single-copy genes. Comparative phylogenetic analysis of the GH family 31 was done by downloading the seed alignment from the pfam database, aligning the sequences using MAFFT [52], and then adding 219 putative novel YihQ/SftG sequences (70 characterized GH family 31 proteins, 70 hits from the Uniprot database using the YihQ/SftG HMM, and 79 unique YihQ/SftG homologs from the 83 genomes encoding the SFT pathway) and 8,696 GH family 31 sequences. For comparative phylogenetic analysis of pyruvate formate-lyase like enzymes, we retrieved amino acid sequences of known choline trimethylamine-lyases, 4-hydroxyphenylacetate decarboxylases, hydroxyproline dehydratases, aryl- alkyl-succinate synthases, glycerol dehydratases, and pyruvate formate-lyases from the KEGG ligand enzyme nomenclature database and clustered them at 90% identity using usearch [53]. Twenty-three glycyl radical enzyme sequences, including known isethionate sulfite-lyases, from genomes of human-gut *Desulfovibrionaceae* were added to the 1204 reference amino acid sequences. Approximate maximum likelihood trees were constructed from alignments with FastTree2 using the JTT + CAT model.

## Results and discussion

### SQ is cooperatively metabolized *in fimo* mainly by *E. rectale* and *B. wadsworthia* to acetate and H_2_S

**Fig. 1:**
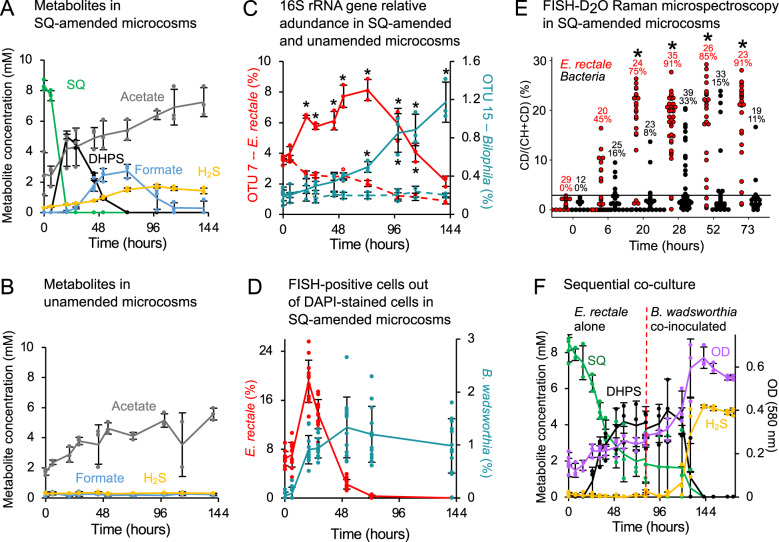
Sulfoquinovose is anaerobically degraded to H_2_S by DHPS-cross-feeding between *Eubacterium rectale* and *Bilophila wadsworthia*. **A** Degradation of SQ (green) in anoxic human fecal microcosms resulted in transient production of DHPS (black) and formate (blue) and accumulation of H_2_S (yellow) and acetate (gray). Formate is a possible electron donor for respiration of DHPS-derived sulfite. **B** Unamended control microcosms did not produce DHPS, formate, or H_2_S. **C** Relative abundance of *E. rectale* (red) and *B. wadsworthia* (teal) 16S rRNA gene OTUs in microcosms with SQ (solid lines) and without amendment (dashed lines). **D** FISH quantification of *E. rectale* and *B. wadsworthia* in SQ microcosms. Error bars represent one standard deviation of averages from 12 microscopic fields. **E** FISH-Raman analysis of single-cell activity (%CD labeling) of *E. rectale* (red) and *Bacteria* (black) in SQ-amended fecal microcosms containing 50% D_2_O. Numbers indicate the number of cells analyzed and percentages of active cells (above a 3.54 %CD background threshold; black bar) and asterisks indicate that FISH-positive *E. rectale* cells are significantly enriched in CD vs. 0 h (*P* < 0.01). **F** Two-step degradation of SQ via DHPS to H_2_S by a co-culture of *E. rectale* DSM 17629 (grown in pure culture for 81 h) and *B. wadsworthia* 3.1.6 (co-inoculated at 81 h, dotted line). In **A**, **B**, and **C**, lines represent averages of triplicate measures with error bars representing one standard deviation. Asterisks show significant differences (*P* < 0.01) in OTU relative abundance between matched time points of SQ and unamended microcosms. SQ sulfoquinovose, DHPS 2,3-dihydroxypropane-1-sulfonate, OTU operational taxonomic unit, FISH fluorescence in situ hybridization, DAPI 4′,6-diamidino-2-phenylindole, OD optical density at 580 nm.

To investigate SQ degradation by human gut microbiota, we constructed triplicate anoxic microcosms with fecal slurries mixed from eight vegetarians and incubated them with SQ (10 mM) or control substrates (Fig. S1A). SQ was completely consumed within 20 h concomitant with a transient accumulation of DHPS (up to 5 mM) between 20 and 52 h (Fig. 1A). As DHPS was consumed, we observed increased production of H_2_S with a peak at 96 h. In addition, formate accumulated transiently with a peak at 73 h (about 3 mM), while acetate gradually increased over 144 h (to 7.5 mM) (Fig. 1A), compared to unamended control microcosms (Fig. 1B). Primary SQ and secondary DHPS degradation was accompanied by select changes in community composition, with only nine species-level 16S rRNA gene operational taxonomic units (OTUs) increasing significantly (*P* < 0.01) in relative abundance compared to unamended microcosms (Fig. S2A, Tables S4 and S5). Temporal abundance changes of the two most strongly increasing OTUs, identified as *E. rectale* (*Agathobacter rectalis* [40]) and *Bilophila wadsworthia* (Fig. 1C, Fig. S2A), were confirmed by fluorescence probe-based microscopy cell counting (Fig. 1D) and corresponded with the consumption of SQ and DHPS, respectively (Fig. 1A, C). Stable isotope probing of individual cells in SQ- and heavy water (D_2_O)-amended microcosms demonstrated high cellular activity (up to 29% D-labeling) of *E. rectale* (Fig. 1E), suggesting that SQ was utilized for growth [32]. Amplicon sequencing of *dsrB*, encoding the beta-subunit of a key enzyme for H_2_S production, dissimilatory sulfite reductase, revealed a significant increase in a *Bilophila* OTU at 20 h and later (>44 h) also in two *Desulfovibrio* OTUs (*P* < 0.01; Fig. S1I, Tables S6 and S7), in concert with DHPS and H_2_S dynamics (Fig. 1A). Consistently, phylogenomic analysis of 93 MAGs from the SQ-amended microcosm (Fig. 1A and Table S8) identified one MAG as *E. rectale* (SQ_MAG_41, average nucleotide identity (ANI) of >97% to 42 *E. rectale* strains; Tables S8 and S9) and a second MAG as *B. wadsworthia* (SQ_MAG_14, ANI of >97% to three *B. wadsworthia* strains; Tables S8 and S10). While a few other bacteria are likely also involved (Fig. S2A), these data suggest that SQ is cooperatively metabolized in the microcosms mainly by *E. rectale* and *B. wadsworthia* with net production of acetate and H_2_S. We confirmed this finding by co-culturing isolates of *E. rectale* (DSM 17629) and *B. wadsworthia* (strain 3.1.6) with SQ. *E. rectale* grew by fermenting SQ and produced DHPS, which was rapidly consumed after co-inoculation of *B. wadsworthia* leading to near-stoichiometric production of H_2_S (Fig. 1F). The co-culture reproduced the sulfur metabolite dynamics observed in our SQ-amended microcosms and affirms DHPS-cross-feeding between *E. rectale*, one of the most abundant gut microbiota members in healthy individuals [54, 55], and *B. wadsworthia*, a pathobiont associated with gastrointestinal inflammation and cancer [21, 26, 56, 57].

We next applied genome-resolved metatranscriptomics on the fecal microcosms and differential proteomics on pure-culture experiments to identify the genes/proteins involved in complete catabolism of SQ to H_2_S.

### *E. rectale* ferments SQ in the gut via the transaldolase pathway to produce DHPS

**Fig. 2:**
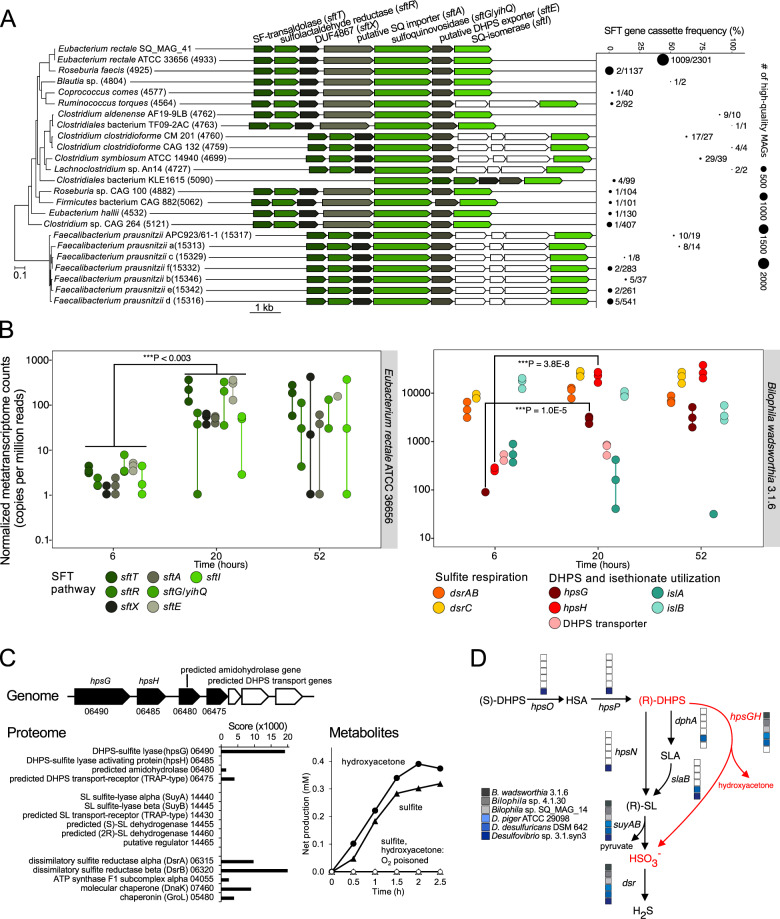
Distribution, composition, and expression of genes involved in sulfoquinovose degradation by *Eubacterium rectale* and DHPS degradation by *Bilophila wadsworthia*. **A** Structure of the SQ-utilization gene cluster for SQ utilization via the SFT pathway in human gut bacteria and the *E. rectale* SQ_MAG_41 recovered from the microcosms. Genes depicted in white have no predicted function. The four core genes of the SFT pathway (in green shades) are present in 23 species-level MAG clusters (of 4,930 total clusters; each >95% MAG ANI) that are *Lachnospiraceae* and *Ruminococcaceae* family members. MAG clusters are ordered based on the phylogeny of representative genomes. Numbers in parentheses denote MAG species-cluster IDs [45]. SQ_MAG_41 is a member of the *E. rectale* ATCC 33656 species cluster. SFT gene cassette frequency across each species-level MAG cluster with point labels indicating the number of MAGs containing the SQ-gene cassette with respect to the total number of MAGs in each species-level cluster. **B** Expression of pathways for SQ utilization by *E. rectale* (transcripts mapped to *E. rectale* ATCC 36656) (left) and DHPS utilization and sulfite respiration by *B. wadsworthia* (transcripts mapped to *B. wadsworthia* 3.1.6) (right) in the metatranscriptomes of triplicate (*n* = 3) fecal microcosms at 6, 20, and 52 h after amendment with 10 mM SQ. Vertical lines connect non-overlapping replicate data points and asterisks indicate significant difference in expression level. DUF domain of unknown function, SF 6-deoxy-6-sulfofructose, SQ sulfoquinovose, DHPS 2,3-dihydroxypropane-1-sulfonate, HSA 2-oxo-3-hydroxy-propane-1-sulfonate, SLA 3-sulfolactaldehyde, SL 3-sulfolactate. **C** Structure of the DHPS utilization gene cluster in *B. wadsworthia* 3.1.6 and its inducibly expressed proteins (bar graph) during growth with DHPS as electron acceptor (numbers refer to RefSeq locus tag numbers; prefix HMPREF0179_RS) (Fig. S5C shows a comparison to growth with taurine, isethionate, and 3-sulfolactate as electron acceptors). Metabolite analysis (line graph) of cell-free extracts of DHPS-grown cells indicated cleavage of DHPS into sulfite and hydroxyacetone, if the reaction was performed under strictly anoxic conditions [28]; representative results (*n* = 5). **D** Presence/absence of DHPS-utilization pathway genes in *B. wadsworthia* genomes and other selected *dsrABC*-encoding human gut *Desulfovibrionaceae*.

*E. rectale* SQ_MAG_41 was the only MAG from the SQ-amended microcosms metagenome that contained a cluster with SQ metabolism genes and *yihQ/sftG* (Fig. 2A), which is co-located with all previously characterized SQ-degradation gene clusters [12, 13, 15] and encodes a sulfoquinovosidase (*alpha*-glucosidase) that cleaves SQ from SQDG/SQG [14, 44]. All sulfoquinovosidase homologs form a separate monophyletic lineage within the GH family 31 tree (Fig. S3). Transcription of the *E. rectale yihQ/sftG* as well as of 10 flanking genes was significantly upregulated upon SQ degradation in the microcosms (*P* < 0.02, Fig. 2B, Table S11). We independently confirmed co-expression of these 11 co-localized genes by constructing a gene expression network for *E. rectale* using publicly available metatranscriptomes from 1090 human fecal samples [58–60] (Fig. S4). Besides *yihQ/sftG*, this cluster includes all genes of the SFT pathway that was active during SQ fermentation by *E. rectale* strain DSM 17629 [15]. Here, an SQ isomerase (SftI) converts SQ to 6-deoxy-6-sulfofructose. A transaldolase (SftT) then catalyzes interconversion of 6-deoxy-6-sulfofructose with glyceraldehyde-3-phosphate to 3-sulfolactaldehyde and fructose-6-phosphate. Fructose-6-phosphate is funneled into glycolysis for fermentation and growth. 3-Sulfolactaldehyde is reduced to DHPS by an NADH-dependent reductase (SftR) as an additional fermentation step [15], and the DHPS is exported via SftE.

We subsequently queried 73,793 high-quality MAGs, assembled from 9,428 human gut metagenomes [45], for the four core catalytic enzyme genes (*yihQ/sftG*, *sftI*, *sftT*, *sftR*) and found a surprisingly narrow distribution of the SFT pathway across human gut species. All four genes were present in only 23 (0.5%) of 4,930 species-level genome bins [45] and mostly at low frequency (Fig. 2A). A notable exception is the *E. rectale* genome bin with 44% of its 2,301 MAGs encoding the SFT pathway (Fig. 2A), suggesting that *E. rectale* is the most prevalent SQ-metabolizing organism in the human gut.

### *B. wadsworthia* desulfonates DHPS to sulfite via an oxygen-sensitive, glycyl radical DHPS sulfite-lyase

At the start of our study, DHPS was an unknown substrate for *B. wadsworthia* to generate H_2_S (Fig. S5A) and we thus sought to identify the genes responsible for DHPS catabolism, such as genes for enzymes converting DHPS to 3-sulfolactate, the substrate for the desulfonating enzyme SuyAB [17]. Screening the genomes of human gut-associated *Desulfovibrionaceae* for known genes involved in the metabolism of DHPS and other organosulfonates for respiration of sulfite (Fig. S5B) [17, 28] revealed that the currently described genes for linking DHPS catabolism to the 3-sulfolactate desulfonation pathways (the DHPS dehydrogenase genes *hpsO* and *dhpA*) [17, 61] are absent in all known *Bilophila* genomes, indicating that DHPS might not be metabolized via 3-sulfolactate in *B. wadsworthia*. Instead, we identified for the pure culture of *B. wadsworthia* 3.1.6 through differential proteomics that it strongly and specifically expresses a four-gene cluster during growth with DHPS. This cluster includes genes for a new glycyl radical DHPS sulfite-lyase system (HpsGH) (Fig. 2C, D, Fig. S5C–E) that was recently identified and characterized [18]. HpsG is a closely related but phylogenetically distinct paralog of isethionate (2-sulfoethanol) sulfite-lyase IslA [28] (also designated IseG [62]) (60.6% identity to HpsG) in *B. wadsworthia* (Fig. S6). IslA catalyzes isethionate desulfonation, a key reaction in taurine degradation [28, 62]. HpsH is the activator of HpsG [18], analogous to the IslA-activating, radical SAM enzyme IslB [28]. Accordingly, *hpsG* and *hpsH* were the most differentially expressed *B. wadsworthia* genes in the SQ-degrading fecal microcosms (n-fold change of 83 and 93, respectively; *P* = 3.8 × 10^−8^ and 1.0 × 10^−5^, respectively, Fig. 2B, Table S12). Highly oxygen-sensitive glycyl radical enzymes are a diverse and abundant protein family of the human gut microbiota, yet largely remain functionally uncharacterized [63]. In accordance with findings by Liu et al. [18], DHPS was desulfonated into sulfite and hydroxyacetone by HpsGH (Fig. S5E) in cell-free extracts of DHPS-grown *B. wadsworthia* cells, but only when assayed under strictly anoxic conditions (Fig. 2C), similar to the highly oxygen-sensitive IslA reaction [28, 62]. Furthermore, the pure culture excreted hydroxyacetone during DHPS degradation (Fig. S5A). While IslA cannot utilize DHPS as a substrate [28], HpsGH has a ~100-fold preference for DHPS vs. isethionate [18]. *B. wadsworthia* thus uses HpsG, a glycyl radical C–S bond-cleaving DHPS sulfite-lyase, to produce sulfite from DHPS intracellularly, which is then respired to H_2_S via the DsrAB-DsrC-dissimilatory sulfite reductase pathway (Fig. 2D) [64, 65]. Collectively, we show that, in a human fecal microbiota, *E. rectale* and *B. wadsworthia* each employ recently discovered metabolic pathways to engage in interspecies DHPS transfer for joint degradation of plant diet-derived SQ to H_2_S.

### Active SQ fermentation and H_2_S production across humans with various gut health states

**Fig. 3:**
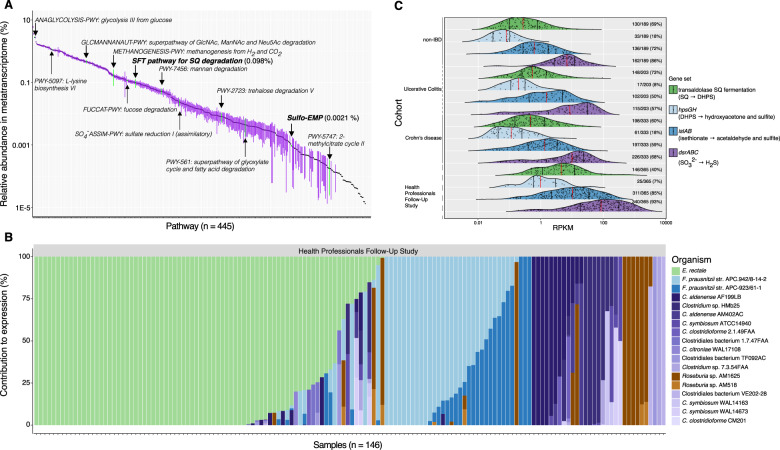
Expression of microbial pathways for SQ fermentation and H_2_S production are abundantly and frequently detected in human stool metatranscriptomes. **A** Ranked relative abundance of 445 identified pathways in 1,090 human stool metatranscriptomes from 201 individuals; each point is the mean relative abundance of a pathway and error bars correspond to the 95% confidence interval of the mean. **B** Relative % contribution of various *Firmicutes* (depicted in various colors) to expression of the SFT pathway in the HPFS cohort (*n* = 146). Each bar corresponds to one sample. See “Materials and methods” for references. **C** Expression of the SFT pathway, DHPS and isethionate utilization (*hpsGH* and *islAB*, respectively), and sulfite respiration (*dsrABC*) in 1,090 human stool metatranscriptomes from 201 individuals from 4 cohorts. The height and area of the ridgeline plots correspond to the distribution of expression. Each point corresponds to 1 sample, the red vertical lines depict the median, and the black lines correspond to the 25th and 75th percentiles. The number of samples with detectable expression within each group is indicated to the right of each plot. HPFS Health Professionals Follow-up Study, RPKM reads per kilobase per million reads, SQ sulfoquinovose, DHPS 2,3-dihydroxypropane-1-sulfonate.

To explore how widely distributed and active SQDG/SQ metabolism is in the human gut, we analyzed the expression of the SFT pathway, and *hpsGH*, *islAB*, and *dsrABC* in the 1,090 stool metatranscriptomes from the Health Professionals Follow-Up Study (365 samples from 96 healthy men) [58] and an inflammatory bowel disease (IBD) study (189, 203, and 333 samples from 27 healthy individuals, 28 with ulcerative colitis, and 50 with Crohn’s disease, respectively) [60]. Consistent with previous reports [58, 60, 66], glycolysis (from glucose and glucose-6-phosphate) and starch degradation were in the top 5% of 445 expressed microbial pathways across all datasets (Fig. 3A). We found that the SFT pathway was within the top third (136th) of all 445 expressed pathways and transcribed prevalently among individuals across all cohorts (177 out of 201 individuals). Further underlining the importance of SQ as a microbial nutrient in the gut, mean SFT pathway transcription was at a similar level to pathways for the usage of fucose (125th) and N-acetylneuraminate (149th) that are abundant components of glycoproteins and glycolipids in the colonic epithelium (Fig. 3A). Contrary to diet-derived SQ, these host-derived sugars represent permanently available substrates for microorganisms in the gut [7]. Furthermore, we determined that the mean relative abundance of the SFT pathway was two orders of magnitude higher than the proteobacterial sulfo-EMP pathway (321st), which was actively expressed in only 28 samples (Fig. 3A). In contrast to what has been hypothesized [17], *E. coli* or other *Enterobacteriaceae* were only minor contributors to SQ-degradation pathway expression, as assessed from fecal samples of these cohorts. We further found that in many individual samples (51%) only a single bacterial species was responsible for more than 50% of SFT pathway transcription. Eighteen putative SQ degraders of the families *Lachnospiraceae* and *Ruminococcaceae* (*Firmicutes*) expressed the SFT pathway (Fig. 3B). *E. rectale*, *Faecalibacterium prausnitzii*, *Clostridium aldenense*, *Roseburia* sp. AM16-25, and *Clostridium clostridioforme* contributed most to expression of the SFT pathway but *E. rectale* was the single most dominant species across both healthy individuals (Fig. 3B) and those with ulcerative colitis or Crohn’s disease (276, 148, and 198 metatranscriptomes, respectively) (Fig. S7). The reasons for these species-specific SFT pathway expression patterns across individual fecal samples are unknown, but might depend on the dynamic intestinal concentrations of SQ and the possibly different kinetics of SQ-degrading enzymes of different microorganisms.

Genes for the DHPS sulfite-lyase (*hpsG*) and its activating enzyme (*hpsH*) were also expressed in human stool (136 positive samples out of 1,090). Samples with detectable expression of the SFT pathway (*n* = 622) did have a higher proportion of samples showing expression of the DHPS pathway (15.6% or 97 of 622 samples) when compared to those samples without detectable SFT pathway expression (8.3% or 39 of 468 samples, Fig. 3C, Fig S7). However, this was not statistically significant and the asynchronous consumption of SQ and DHPS in the fecal microcosm incubations (Fig. 1A) suggests that expression of these pathways is also time-delayed. Furthermore, the isethionate sulfite-lyase genes (*islAB*) that enable *B. wadsworthia* to utilize the sulfite from taurine and isethionate as an electron acceptor [28] were transcribed more prevalently (736 samples) and at a higher relative abundance when compared to *hpsGH* (Fig. 3C). Through meat consumption and microbial deconjugation of host-secreted taurocholic bile acids [2, 56, 67], taurine is a continuously available substrate in the gut and thus could decouple the DHPS-mediated physiological interaction of *B. wadsworthia* with the primary SQ fermenters. Transcription of the DsrAB-DsrC pathway for H_2_S production was abundant and prevalent in the dataset (843 positive samples out of 1,090), and largely dominated by *B. wadsworthia* (Fig. 3C, Fig. S7). Notably, we did not detect significant differences in expression of SQ degradation and H_2_S production pathways between cohorts of IBD patients and healthy individuals, which is likely because, as reported [66], stool samples were not selectively taken from patients with active IBD and thus, contrary to previous studies [68, 69], the observed species composition in stool was not significantly different between IBD and control cohorts. Overall, our re-analysis of stool metatranscriptomes from the four cohorts highlighted *B. wadsworthia* as a major sulfidogen and DHPS as an additional substrate for microbial H_2_S production in the human gut. *B. wadsworthia* is specialized in utilizing diverse organosulfonates that are energetically more favorable compared to sulfate for sulfite-respiring bacteria [17, 28, 70].

## Conclusions

**Fig. 4:**
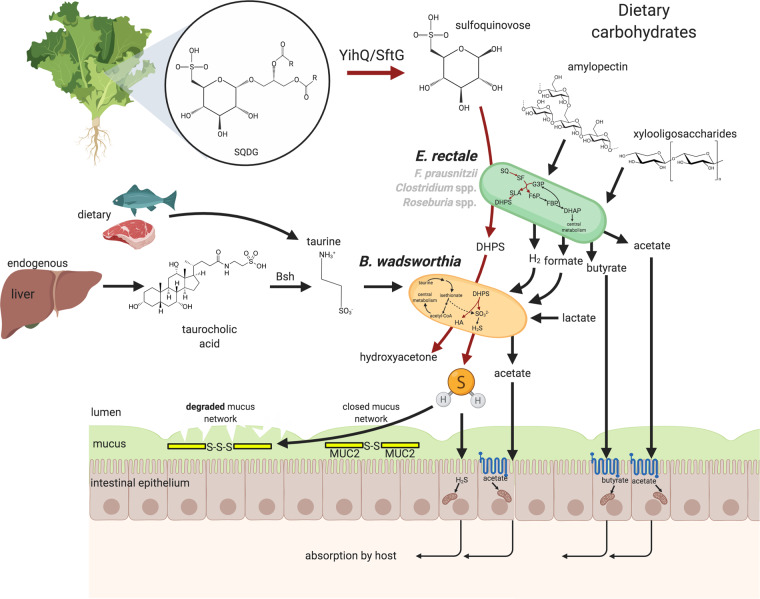
Sulfur energy metabolism and physiological interaction scheme of *E. rectale* and *B. wadsworthia* in the human gut. SQDG present in green vegetables enters the intestine as a common component of the human diet. Sulfoquinovosidases [14], encoded by *yihQ*/*sftG*, cleave the glycosidic linkage of the lipid tail to liberate SQ, which is then in most individuals fermented mainly by *E. rectale* to DHPS, acetate, and likely also to formate or H_2_ and CO_2_. Further plant-derived compounds, such as amylopectin, xylooligosaccharides and other carbohydrates, may also be catabolized and fermented by *E. rectale*. Other *Firmicutes* with the SFT pathway (grey) such as members of the genera *Faecalibacterium*, *Roseburia*, and *Clostridium* can also function as primary SQ degraders in some individuals. Some *Proteobacteria* such as *E. coli* may catabolize SQ via the sulfo-EMP pathway. *B. wadsworthia* cleaves the C–S bond in DHPS via a DHPS-sulfite lyase to produce sulfite for dissimilatory reduction to H_2_S gas coupled to oxidation of, e.g., formate, lactate, and hydrogen, while hydroxyacetone and also acetate are excreted. In few individuals, also *Desulfovibrio piger* uses DHPS as substrate for anaerobic sulfite respiration. Taurine is present in dietary meat/fish and is liberated from host-secreted taurocholic acids by microbial bile salt hydrolases (encoded by *bsh*), and thus far was considered the sole major source of sulfonate-sulfur for respiration and H_2_S production by *B. wadsworthia* in the human intestine. H_2_S and short-chain fatty acids are utilized by colonic epithelial cells as energy sources, act as signaling molecules, and are absorbed and further distributed by the host via the bloodstream [72–74]. Excessive concentrations of H_2_S gas can break the mucus layer in the colon by reduction of Muc2-mucin disulfide bonds to trisulfides [24], thereby allowing bacteria to penetrate the mucus layer and interact with the host epithelial lining. Arrows colored in black represent previously described metabolic pathways, while those in dark red represent pathways revealed in this study. SQDG sulfoquinovosyl diacylglycerol, SQ sulfoquinovose (6-deoxy-6-sulfoglucose), SF 6-deoxy-6-sulfofructose, G3P glyceraldehyde-3-phosphate, F6P fructose-6-phosphate, FBP fructose-1,6-bisphosphate, DHAP dihydroxyacetone phosphate, SLA 3-sulfolactaldehyde, DHPS 2,3-dihydroxypropane-1-sulfonate, HA hydroxyacetone. This figure was created using Biorender.com.

By uncovering the activities of genes for degradation of the plant-derived sulfonated monosaccharide SQ in the human gut microbiota, we have shown that SQ is an exclusive substrate for only a few gut microorganisms, particularly the abundant *E. rectale*. The concept of exclusive nutrient access [5] promises SQ dosage-dependent control over the abundances and activities of these bacteria that are generally associated with a positive impact on human health [3, 71]. We have also demonstrated that interspecies transfer of the SQ-degradation product DHPS is a previously unknown physiological link between a plant-based diet and H_2_S production by the intestinal pathobiont *B. wadsworthia*, which uses DHPS in addition to taurine (Fig. 4). H_2_S is an important intestinal metabolite that influences the activity of epithelial and microbial cells and has beneficial as well as detrimental effects on the colonic environment [24, 27]. The complex host-endogenous and microbial processes regulating intestinal H_2_S homeostasis and how they are influenced by diet or health state are insufficiently defined [20, 27]. Our work emphasizes the roles of microbial cooperation and sulfur metabolism in the human intestinal tract and uncovers new physiological and genetic features of prevalent microbiota members that will inform dietary and drug-based therapies [27] for targeted modification of microbiota membership and H_2_S levels in intestinal disease.

## Supplementary information

Supplementary Information

Supplementary Tables S1-S12

Supplementary File S1

## Data Availability

All sequence data generated in this project is available at NCBI under BioProject PRJNA593787.
