# Supplementary material for: Sulfoquinovose is a select nutrient of prominent bacteria and a source of hydrogen sulfide in the human gut
Source: ISME J. 2021 Mar 31;15(9):2779–91. doi: 10.1038/s41396-021-00968-0 (PMC8397734; doi:10.1038/s41396-021-00968-0)
Supplement: Supplementary file 1 — Supplementary Information [file 41396_2021_968_MOESM1_ESM.pdf]

Sulfoquinovose is a select nutrient of prominent bacteria and a source of hydrogen sulfide in the human gut

Buck T. Hanson, K. Dimitri Kits, Jessica Löffler, Anna G. Burrichter, Alexander Fiedler, Karin Denger, Benjamin Frommeyer, Craig W. Herbold, Thomas Rattei, Nicolai Karcher, Nicola Segata, David Schleheck, and Alexander Loy

Supplementary Information

Contents

A. **Supplementary Materials and Methods**
B. **Supplementary Figures**
Fig. S1. Sulfoquinovose is rapidly degraded in anoxic microcosms with human feces.
Fig. S2. Addition of sulfoquinovose to human feces microcosms triggers only minor shifts in microbiota composition.
Fig. S3. Comparative phylogenetic analysis of glycoside hydrolase family 31 enzymes, including YihQ/SftG sulfoquinovosidases.
Fig. S4. A genome-wide *E. rectale* transcription network reveals co-expression of a sulfoquinovosidase gene-containing gene cluster in human fecal samples.
Fig. S5. Identification of a DHPS-desulfonating enzyme (HpsG) in *B. wadsworthia* 3.1.6 and its distribution among selected sulfur compound-metabolising human gut bacteria.
Fig. S6. Genes for the DHPS sulfite-lyase (*hpsG*) and isethionate sulfite-lyase (*islA*) of *B. wadsworthia* are paralogous.
Fig. S7. *Eubacterium rectale* and *Bilophila wadsworthia* are the dominant contributors to expression of the SFT fermentation and H_2S production pathways in human stool metatranscriptomes, respectively.
C. **Supplementary Tables**
Table S1. 16S rRNA-targeted oligonucleotide probes used for fluorescence *in situ* hybridization.
Table S2. SFT pathway-encoding bacterial genomes in the NCBI database.
Table S3. SFT pathway-encoding genomes/MAGs from a MAG/genome database of human gut microorganisms.
Table S4. 16S rRNA gene amplicon OTU table.
Table S5. Results of 16S rRNA gene DESeq2 analyses.
Table S6. *dsrB* amplicon OTU table.
Table S7. Results of *dsrB* DESeq2 analyses.
Table S8. MAGs from human fecal microcosms.
Table S9. *E. rectale* genome average nucleotide identity (ANI) matrix.
Table S10. *B. wadsworthia* genome ANI matrix.

Table S11. Significance values for *E. rectale* transcripts expressed in microcosms.

Table S12. Significance values for *B. wadsworthia* transcripts expressed in microcosms.

D. **Supplementary Files**

File S1. File S1 YihQ-SftG.hmm

E. **Supplementary References**

A. Supplementary Materials and Methods

Human fecal microcosms. Human fecal samples were collected from healthy individuals adhering to ovo-lacto-vegetarian diets and who had not received antibiotics in the prior 6 months. Sampling and microbiota analysis of human fecal samples was approved by the University of Vienna Ethics Committee (reference #00161). Study participants provided informed consent and self-sampled using an adhesive paper-based feces catcher (FecesCatcher, Tag Hemi, Zeijen, NL) and a sterile 107 x 25 mm polypropylene tube with a screw-cap-attached sampling spoon (Sarstedt, Nümbrecht, DE). Samples were kept cool with ice during transport periods and stored at 4°C for up to a 48 h period. Feces from eight individuals were transferred to an anaerobic chamber (Coy, Grass Lake, MI) having a mixed-gas atmosphere of 5% H_2, 10% CO_2, 85% N_2 (Air Liquide, Austria). Fecal materials were pooled and a final mass of 3.9 g was homogenized in 30 mM anaerobic bicarbonate buffer, pH 6.8. Fecal homogenate was distributed into 20 ml hungate tubes across five treatment conditions in triplicate including buffer alone, 10 mM glucose, 10 mM taurine with 10 mM formate, 10 mM SQ, and 10 mM SQ with 50% D_2O (99.9 atom % D, Sigma-Aldrich) having a final volume of 10 ml with a fecal concentration of 19.5 mg/ml. The concentrations of SQ in the human intestinal tract are unknown. To provide some context, we have calculated the amount of SQ that is in spinach, which contains 824 µg SQDG/g dried plant material [1]. With a molecular weight of 834.2 and 244.2 g/mol for SQDG and SQ, respectively, SQ is present at 0.24 mg/g dried spinach. A gram of fresh spinach (~91% water content) contains 0.022 mg SQ. Each microcosm (10 ml of 10 mM SQ) contained 24.4 mg SQ. Tubes were sealed with butyl-rubber stoppers and screw-on caps and incubated at 37°C. One ml of each incubation was subsampled at 0, 6, 20, 28, 44, 52, 73, 99, 114, and 140 h for metabolite and nucleic acid analyses. Twenty µl of each subsample was added to 100 µl of 2 g/l zinc acetate for fixation of H_2S. Two-hundred µl were fixed with paraformaldehyde (PFA) for FISH-based analyses as described previously [2, 3]. The remaining sample volume was centrifuged at 20,000 X *g* at 4°C to pellet biomass and stored at -80°C for nucleic acid extraction. For SQ and DHPS quantification, 500 µl of the biomass-free supernatant was transferred to a 2 ml glass vial containing 170 µl acetonitrile (Sigma-Aldrich, Austria) and crimp-sealed with butyl-rubber

stoppers and stored at -20°C. The remaining supernatant (~300 µl) was transferred to a new microcentrifuge tube for quantification of anionic metabolites (e.g. short-chain fatty acids).

Pure culture and defined co-culture experiments. *E. rectale* DSM 17629 (purchased from the Leibniz Institute DSMZ-German Collection of Microorganisms and Cell Cultures, Braunschweig, Germany) and *B. wadsworthia* strain 3.1.6 (kindly provided by Dr. Emma Allen-Vercoe, University of Guelph, Canada) were grown under conditions as described previously [4–6]. Briefly, a carbonate-buffered mineral-salts medium reduced with titanium(III)-nitriloacetate (Ti(III)-NTA) was used under a N_2/CO_2 gas phase (80:20) in butyl-rubber stoppered serum flasks (20-ml scale) or culture tubes (10-ml scale). All cultures were incubated at 37°C in the dark without shaking. For growth of *E. rectale* with SQ or glucose as substrates for fermentation, the medium was supplemented with yeast extract (0.1% *w*/*v*), hemin (0.5 µg/ml) and vitamin K1 (0.1 µg/ml). For growth of *B. wadsworthia* with DHPS, taurine, 3-sulfolactate, or isethionate as electron acceptors (10 mM each), the medium was supplemented with 1,4-naphthochinone (0.2 µg/ml) and with lactate (20 mM) (or formate) as an electron donor. At intervals, growth was monitored as optical density (OD_{580nm}) of the resuspended cultures (after mixing by inversion), of which samples were taken to determine sulfonate disappearance and formation of products (see below). Cells were harvested from *B. wadsworthia* 3.1.6 replicate growth experiments in the late exponential growth phase for differential proteomics when grown with the four different organosulfonates (Fig. S5c) or for preparation of anoxic cell-free extracts for DHPS sulfite-lyase enzyme tests (Fig. 2c) (described below). For co-cultivation experiments, medium containing SQ as the sole substrate for fermentation and organosulfonate respiration plus all supplements (above) was used and either both strains were co-inoculated at the start of the growth experiment or (Fig. 1f) *E. rectale* DSM 17629 was inoculated first (n=3) and after 81 hours *B. wadsworthia* 3.1.6 was co-inoculated.

Anoxic cell-free extracts and enzyme tests. Preparation of anoxic cell-free extracts of *B. wadsworthia* 3.1.6 and DHPS sulfite-lyase assays were done under conditions as described previously for the isethionate sulfite-lyase (IslA) reaction [4]. Briefly, cultures were harvested in the late exponential growth phase (OD_{580} appox. 0.3 – 0.4) by centrifugation of the serum bottles. Cells were resuspended in anoxic Tris-HCl buffer (50 mM, pH 8.0) containing $MgCl_2$ (5 mM) and disrupted by three passages through a cooled French pressure cell, which had been flushed with N_2 gas. The enzyme was assayed discontinuously at room temperature by monitoring the formation of the two products sulfite and hydroxyacetone. The reaction mixture (1 ml) contained 20 mM DHPS, 1 mM *S*-adenosylmethionine chloride (SAM), 1 mM Ti(III)-NTA, and about 0.2 – 0.9 mg total protein in 50 mM Tris-HCl (pH 8.0) containing $MgCl_2$ (5 mM). The buffer, including DHPS

and SAM, was initially degassed under vacuum in glass cuvettes with rubber stoppers, and flushed with nitrogen gas, three times each. Then, Ti(III)-NTA was added, and the reaction was initiated by the addition of anoxic crude extract, each with a syringe and needle through the rubber stoppers. At appropriate time intervals, samples (100 µl) were taken by syringes for quantification of sulfite by a colorimetric assay and of hydroxyacetone by derivatization and HPLC-UV analysis (see below).

Differential proteomics. Total proteomic analyses of cell-free extracts of *B. wadsworthia* 3.1.6 were done as previously described [4] at the Proteomics Centre of the University of Konstanz (https://www.biologie.uni-konstanz.de/proteomics-centre/). Briefly, each trypsin-digested and purified sample was analyzed twice on a Orbitrap Fusion with EASY-nLC 1200 (Thermo Fisher Scientific), and tandem mass spectra were searched against the protein database using Mascot (Matrix Science) and Proteome Discoverer v1.3 (Thermo Fisher Scientific) with "Trypsin" enzyme cleavage, static cysteine alkylation by chloroacetamide, and variable methionine oxidation.

Metabolite analyses***.*** H_2S was quantified colorimetrically [7]. Absorbances at 670 nm were measured on a transparent 96-well plate (Greiner Bio-One, Austria) using an Infinite 200 PRO spectrophotometric microplate reader (TECAN Group Ltd., Männedorf, Switzerland). The samples were quantified with external standards prepared in parallel using sodium sulfide (Sigma-Aldrich, St. Louis, Missouri, USA). Biomass-free supernatants were subjected to capillary electrophoresis for separation and quantification of short-chain fatty acids on a P/ACE–MDQ apparatus (Beckman Coulter, Krefeld, Germany) equipped with a UV-detector with a wavelength filter at 230 nm. The capillary was a fused silica column (TSP075375; 75 µm ID, Polymicro Technologies) 60 cm long (50 cm to the detector) and 75 µm in diameter. Samples were processed using a CEofix Anions 5 Kit (Beckmann Coulter, Krefeld, Germany) according to the manufacturer's instructions. Analytes were separated in reverse polarity mode at 30 kV (ramp: 0.5 kV/s) for 10 min. All samples were diluted 1:10 with a working solution consisting of 0.01 M NaOH, 0.5 mM $CaCl_2$ and 0.1 mM caproate (internal standard). Analytes were quantified using an external standard mixture of SQ, Na_2SO_4, formate, succinate, acetate, lactate, propionate, butyrate, and valerate. SQ, DHPS, 3-sulfolactate, taurine and isethionate were quantified in culture experiments by HPLC against authentic standards, as described previously [8], using a hydrophilic interaction ZIC-HILIC (Merck, Darmstadt, Germany) column and an evaporative light scattering detector. Hydroxyacetone, short-chain fatty acids, and alcohols in culture experiments were analyzed by a HPLC method described previously [5], using an Aminex column (BioRad) and a refractive index detector. Hydroxyacetone was quantified also by derivatization with dinitrophenylhydrazine and HPLC-UV, using a method described for acetaldehyde [4].

Sulfite in enzyme reactions was quantified using a colorimetric assay (Fuchsin assay) or by derivatization with *N*-(9-acridinyl)maleimide and HPLC-UV [4].

Fluorescence cell counting. FISH probes targeting *E. rectale* (EREC996-Cy5, EREC1252-Cy5) and *B. wadsworthia* (BWA829-Cy3) were designed using ARB [9] and evaluated using SILVA TestProbe 3.0 against the SSU REF database (Release 132; December 13, 2017) (Table S1) [10]. Optimal hybridization conditions were evaluated for fluorescently labeled probes with formamide concentrations ranging 0-70% using PFA-fixed cells of pure cultures of *E. rectale* DSM 17629 and *B. wadsworthia* DSM 11045 (purchased from the Leibniz Institute DSMZ-German Collection of Microorganisms and Cell Cultures, Braunschweig, Germany). For microcosm cell counts, aliquots of PFA-fixed cells (5 or 20 µl) were filtered onto black polycarbonate filters (0.2 µm pore size, GTBP Isopore Membrane Filters, Millipore, Cork, Ireland) using a 25 mm glass vacuum filter holder (Sartorius Stedim Biotech, Goettingen, Germany). One-sixth filter sections were cut and stained with FISH probes using standard procedures [2]. Samples were stained with DAPI (1 µg/mL; Sigma– Aldrich) for 5 min and washed with deionized water. Filter sections were placed on a glass microscope slide and overlain with one drop of Citifluor AF1 (Electron Microscopy Services, Hatfield, PA) and a coverslip. Cells were imaged using a Zeiss Axioplan 2 equipped with a Plan-Neofluar 100 x oil objective with a 1.3 numerical aperture (Zeiss, Germany). Cells from twelve fields of view were manually counted in a grid measuring 0.1225 mm^2.

Single-cell stable-isotope probing by FISH-Raman microspectroscopy. PFA-fixed cells (5-10 µl) from fecal microcosms incubated with 10 mM SQ and 50% D_2O were diluted into 100 µl of 1X PBS and sonicated at 35 kHz for 2 min at 20°C (Sonorex, Brandelin Electronic, Berlin, Germany) followed by centrifugation for 20 min at 20°C and 20,913 X *g*. Liquid-based FISH was performed as previously described [11] using FLUOS-labeled probes targeting *E. rectale* and most *Bacteria* (EUB338mix) [12]. Stained cell pellets were resuspended in 5-10 µl sterile filtered water. One µl of a 1:10 dilution of each sample was spotted onto an aluminum-coated glass slide (Al136; EMF Corporation) and allowed to air dry, followed by a 2 s wash in cold sterile filtered water. After air drying, fluorescence was used to identify cells for Raman analyses. Single cell Raman spectra were acquired using a LabRAM HR800 confocal Raman microscope (Horiba Jobin-Yvon) equipped with a 532 nm neodymium-yttrium aluminum garnet laser and a 300 grooves/mm diffraction grating. D-labeling (%CD) was quantified from integrated C-D and C-H peak areas (2,040-2,300 and 2,800-3,100 cm^{-1}, respectively) as previously described [11]. Cells having %CD values greater than the average plus 3X the standard deviation of unlabeled cells, measured at the 0 h time point (threshold 3.54% CD), were considered D-labeled and metabolically active. Significant enrichment of %CD-labeling in FISH-

positive *E. rectale* cells was evaluated using a Wilcoxon rank-sum test comparing FISH-positive cell counts for each time point with the 0 h time point.

Extraction of nucleic acids. DNA for amplicon and metagenome sequencing and RNA for metatranscriptome sequencing were extracted following standard procedures [13] that included bead beating for 30 s on dry ice using a Lysing Matrix E tube (MP Biomedicals). Purified nucleic acid pellets were air dried at ambient room conditions and resuspended in 30 µl DNase/RNase-free H_2O (Carl Roth GmbH, Germany) and stored at -80°C.

16S rRNA gene and *dsrB* amplicon sequencing. Established two-step PCR barcoding protocols were used for amplicon sequencing of the 16S rRNA gene (with primers H_515F_mod-5'-(head) GCTATGCGCGAGCTGC-GTGYCAGCMGCCGCGGTAA and H_806R_mod-5'-(head) GCTATGCGCGAGCTGC-GGACTACNVGGGTWTCTAAT that target most *Bacteria* and *Archaea*) [14] and the dissimilatory sulfite reductase gene *dsrB* [15]. Blank nucleic acid extractions and negative (water only) PCR reactions were included as controls. Barcoded amplicon libraries were pooled at equivalent copy numbers ($20x10^9$) for 300 bp paired-end MiSeq (Illumina) sequencing Microsynth AG, Balgach, Switzerland). Sequencing results were analyzed according to the procedures outlined previously [14, 15] and clustered into operational taxonomic units (OTU) according to a sequence similarity cut-off of 97% using Uparse [16], which also screens out chimeras. 16S rRNA gene amplicons were classified using RDPclassifier [17] as implemented in Mothur v1.42.3 [18] using Release 132 of the SILVA database. 16S rRNA gene and *dsrB* amplicons that showed significant increases over time in the SQ-amended microcosms were further classified by phylogenetic inference by constructing a maximum likelihood tree (MEGA7 v7.0.18) [19] using a collated dataset of amplicons and their top 10 subjects from querying against the NCBI 16S rRNA reference gene and nr/nt databases, respectively. Classification of *dsrB* sequences was also performed with phylogenetic placement using a curated DsrB reference sequence database and corresponding consensus tree [15, 20]. 16S rRNA gene and *dsrB* libraries were analysed using the software package Phyloseq [21] for R Studio v1.0.143 (rstudio.com). Statistical tests between treatment time points were performed with DESeq2 using the variance stabilizing transformation (vst) [22].

Metagenomics. Samples from three time points (28, 73, and 114 h) from the SQ-amended microcosms were chosen for metagenome sequencing. DNA samples were diluted to 0.1 ng/µl in 130 µl and sheared using a Covaris M220 Focused-ultrasonicator Instrument (Covaris, Woburn Massachusetts, USA) to a target length of 350 bp. Library preparation was conducted according to the NEBNext Ultra II DNA Library Prep Kit for Illumina (New England BioLabs Inc., Ipswich, Massachusetts) protocol. Indexing primers were ligated conforming to the NEBNext Multiplex Oligos for Illumina manual (Index

Primers Set 1, New England BioLabs Inc., Ipswich, Massachusetts). Library fragment sizes and purity were screened using a Bioanalyzer 2100 and the High Sensitivity DNA kit (Agilent Technologies, Santa Clara, CA). Samples were pooled for 150 bp paired-end sequencing on a HiSeq 3000/4000 (Illumina) at the Biomedical Sequencing Facility (BSF) of the Research Center for Molecular Medicine (CeMM), Vienna, Austria. Metagenomic datasets consisted of 87, 93, and 158 M reads (28, 73, and 114 h time points, respectively). For assembly and binning of metagenome-assembled genomes (MAGs), adapters and low-quality base-calls were trimmed from Illumina reads using BBduk (https://jgi.doe.gov/data-and-tools/bbtools/ - right -trimmed, q=15, minlength = 149 bp). Trimmed reads were error-corrected using BayesHammer [23] and assembled using metaSPAdes (SPAdes v3.13.1) [24]. MAGs were binned using MetaBAT v2.12.2 using composition and abundance under all presets on each dataset [25]. MAGs were assessed for quality using checkM v1.0.12 [26] and de-replicated using drep (v1.4.3) [27]. The highly fragmented and incomplete original *Bilophila* sp. MAG_14 MAG was missing *hpsG*, however an unbinned 3913 bp contig (99.3% identity to *B. wadsworthia* 3.1.6) containing the *hpsG* sequence had a similar coverage to the MAG (19.7- vs. 20.4-fold coverage). This contig was tested for consistency with the original MAG through iterative reassembly using MAGspinner v0.10 (github.com/hexaquo/MAGspinner). MAGspinner removes contigs with abnormal coverage and/or tetranucleotide signatures during the reassembly process. The inclusion of the contig containing the *hpsG* sequence in the final bin (30 rounds of reassembly) was interpreted as evidence that the contig belongs to *Bilophila* sp. MAG_14. Representative MAGs were assigned taxonomy through alignment and phylogenetic placement of concatenated marker genes into the Genome Taxonomy Database reference tree (GTDB-Tk) [28]. 16S rRNA gene sequences were extracted from each MAG with nhmmer [29] using rfam [30] models for bacterial and archaeal small subunit rRNAs (RFAM: RF00177, RF01959). Minimum overlap between sequence and model was 300 nucleotides. Sequences were classified using the RDP classifier [17] as implemented in Mothur. The number of tRNAs and associated amino acids were determined using tRNAscan-SE [31]. Pairwise, whole-genome, average nucleotide identity (ANI) between genomes of *Eubacterium rectale* and between genomes of 16 representative *dsrAB*-containing bacteria from the gut, including *Bilophila*, were calculated using FastANI (v1.2) using >95% ANI and <83% as the intra- and inter-species cutoffs [32], respectively.

Metatranscriptomics. Metatranscriptome libraries (n = 9) were prepared from nucleic acid extracts from three timepoints (6, 20, and 52 h) of the triplicate SQ-amended microcosms. DNase digestion of nucleic acid extracts was performed up to three times (ezDNase; Invitrogen, Carlsbad, CA) according to the manufacturer's instructions. Removal of DNA was confirmed using a standard 16S rRNA gene PCR followed by electrophoretic analysis. Total RNA quality was evaluated using a Bioanalyzer 2100 and

Agilent RNA 6000 Pico kit (Agilent Technologies, Santa Clara, CA). rRNA was removed from total RNA using the Ribo-Zero Gold rRNA Removal Kit (Epidemiology) (Illumina Inc., San Diego, CA) according to the manufacturer's instructions. Purified mRNA pools were quantified (Quant-iT RiboGreen RNA Assay Kit, ThermoFisher Scientific) and 1-100 ng was used in each library preparation for multiplexed Illumina RNASeq analysis (NEBNext Ultra RNA Library Prep Kit for Illumina with NEBNext Multiplex Oligos for Illumina primer set 1, New England BioLabs Inc., Ipswich, MA). Indexed libraries were purified from adapter sequences (Agencourt AMPure XP Beads, Beckman Coulter), quantified (Quant-iT PicoGreen RNA Assay Kit, ThermoFisher Scientific), evaluated for size and purity (Bioanalyzer 2100 and the High Sensitivity DNA kit, Agilent Technologies, Santa Clara, CA), and pooled at equimolar contributions. Sequencing (50 bp, single-end) was performed on a HiSeq 3000/4000 (Illumina). The reads were demultiplexed and bam files were converted to fastq using SAMtools (v1.9) [33] followed with trimming using Trimmomatic (v0.39) [34] with the following settings: LEADING:3, TRAILING:3, SLIDINGWINDOW:4:15, MINLEN:40. The trimmed reads were then mapped to the genomes of *Eubacterium rectale* ATCC 33656 (GCF_000020605.1_ASM2060v1) and *Bilophila wadsworthia* 3.1.6 (GCF_000185705.2_Bilo_wads_3_1_6_V2) using bbmap (v37.61; sourceforge.net/projects/bbmap/) with a minid of 97%. Feature counting was done using featureCounts within the Subread package (v1.6.3) [35]; reads that mapped equally well to multiple locations were assigned as fractions of the uniquely mapping reads for the same region (-M --fraction). After removal of reads that mapped to any rRNA, the raw read counts for all libraries were then converted to copies per million counts (cpm) and normalized using a trimmed mean of M-values (TMM) between each pair of samples using calcNormFactors (edgeR, v3.9) [36]. Differential expression analysis was done by pair-wise comparison of the samples from 20 h incubation to the samples from the 6 h incubation using the exactTest function (edgeR, v3.9). Genes were considered differentially expressed if the adjusted p-value was below 0.05 (P < .05).

Construction of a bacterialYihQ/SftG profile hidden markov-model (HMM). Bacterial genomes and MAGs that were publicly available in Genbank as of July 27, 2017 (N=103411) were screened using hmmsearch (v3.1b2) [37] for genes that contained the Glycosyl hydrolases family 31 pfam (PF01055.25). The resulting dataset (N=127608) was further screened using a YihQ/SftG-specific motif that we constructed using the signature sulfoquinovosidase residues reported by Speciale *et al*. [38] corresponding to positions 290-508 in YihQ (Genbank accession AAB03011.1) from *E.coli*: W-X(2)-D-W-X-G-X(6)-G-X(4)-W-X-W-X(1,300)-D-X-G-G-[YWF] (expressed as a PROSITE pattern). Subsequences that matched this motif (N=585, mean length = 225 nt, length standard deviation = 2 nt) were extracted from the full dataset using a simple grep command in a Unix environment and aligned using MAFFT v7.397 [39]. Positions corresponding to the variable-length linker corresponding to positions 308-505 in AAB03011.1 (corresponding

to -X(1,300)- in the prosite pattern) were manually removed from the alignment. Remaining subsequences were clustered at 70% identity using Usearch [40]. Centroid sequences (N=15, length=26) were aligned using MAFFT v7.397. The resulting alignment was used to construct an HMM using hmmbuild (v3.1b2) [37]. The performance of the hmm for identifying the clade of sulfoquinovosidase proposed by Speciale *et al*. [38] was examined phylogenetically. The set of genes that contained the Glycosyl hydrolases family 31 pfam (PF01055.25, N=127608) were length-filtered (min. 50 amino acids) and clustered at 90% identity with Usearch. Centroid sequences (N=2591) were aligned using MAFFT v7.397 and a phylogenetic tree was constructed in FastTree2 [41]. E-values < 1 were diagnostic for the proposed clade of sulfoquinovosidase. Of the 2591 Glycosyl hydrolases family 31 test sequences, 103 belonged to the YihQ/SftG clade and 2488 did not. There were 4 false negatives and 1 false positive, resulting in a balanced accuracy of 0.98.

Genome analyses and comparative genomics. We searched all contigs and MAGs from the SQ-amended microcosm for known SQ metabolism genes using blastp and the YihQ/SftG profile HMM. To identify publicly available genomes that encode the SFT pathway, we searched the NCBI genome database using blastp for organisms that contain all four of the essential genes for the pathway - the sulfoquinovosidase (YihQ/SftG), SQ isomerase (SftI), 6-deoxy-6-sulfofructose transaldolase (SftT), and the SLA reductase (SftR). Each of these genes from the *E. rectale* ATCC 33656 genome was searched against all NCBI genomes using blastp with -max_target_seqs set to 20,000, an e-value cutoff of $1e^{-30}$, and a minimum 70% query coverage. All hits for each gene were then collated, sorted, and cross referenced to identify organisms which had hits for all four genes. Each genome was then checked for co-localization and synteny of the genes of interest to identify those that potentially encoded the complete pathway. This analysis identified 83 bacterial genomes that encode all four core enzymes of the SFT fermentation pathway (Table S2). Additionally, the presence of the SFT gene cluster in 73,798 high-quality MAGs from several thousands of publicly available human gut metagenomes [42], was searched using a two-step approach. Firstly, all translated open reading frames, as inferred with Prokka v1.14.0 [43]/Prodigal v2.6.3 [44], were queried with our YihQ/SftG profile HMM (File S1). Among 1,976 hits, all had an e-value of less than 0.1, and 84% had an e-value of less than 10^{-5}. We did not apply an e-value filter in this step since we expected that the proximity check (see below) removes false positives. Secondly, blast-based searches including the three additional SFT pathway core protein sequences (SftI, SftT, and SftR) were performed against all translated open reading frames of genomes with an YihQ/SftG HMM hit. Physical proximity of the four genes in the genome was checked to validate SQ-gene clusters. We retained blast hits if they were above 75% of the mean reference gene length and had a mean identity of at least 70%. For each query genome and gene, we then kept only that blast hit with the largest e-

value. After these steps, all blast hits had an e-value < 10^{-114}. We then considered only those genomes where all blast hits were found on the same contig and ensured that all of them were within a 10-ORF window. 1118 MAGs passed these filters (Table S3). The reference genome set used for taxonomic annotation of MAG clusters consisted of 80,853 genomes obtained from GenBank (March 2018) corresponding to 17,607 microbial species for which at least one proteome was available in UniProt. 137 isolate genomes [45] were added to this set for a total of 80,990 isolate genomes with confident taxonomic annotation. Concatenated marker gene alignments using representative MAGs from each of the 23 species-level MAG clusters containing the SQ operon were generated using checkM v1.0.12 [26] and placed into a phylogenomic tree using FastTree2 [41]. Genes that enable taurine (*tpa*), DHPS (*hpsGH, hpsO, hpsN, dphA*), 3-sulfolactaldehyde (*slaB*), 3-sulfolactate (*suyAB, slsC, comC*), sulfoacetaldehyde (*xsc, sarD*), and isethionate (*islAB*) catabolism and sulfite reduction (*dsrABC*) were initially identified in genomes of 16 representative *dsrAB*-containing human gut bacteria by blastp screening all protein coding genes from the genomes of interest against custom amino acid sequence databases established for each gene. The blastp results were filtered with an e-value cutoff of $1e^{-30}$ and a minimum 70% query coverage. Neighborhoods of some genes of interest were checked manually for synteny: *tpa* with *sarD*, *adhE* with *islAB*, *dhpA* with *slaB*, *hpsOPN,* and *slsC* with *comC*.

Metatranscriptome analysis of publicly available datasets. Reads from a total of 1,090 paired gut metagenomes/metatranscriptomes were downloaded from https://ibdmdb.org/ (725 paired samples from 105 individuals [46]) and the sequence read archive at https://www.ncbi.nlm.nih.gov/sra (365 samples from 96 individuals, BioProject accession PRJNA354235 [47]. The downloaded reads, which were already screened for reads originating from the host, were then trimmed using Trimmomatic (v0.39) [34] with the following settings: LEADING:3, TRAILING:3, SLIDINGWINDOW:4:15, MINLEN:80. Taxonomic and functional profiling of the metagenomic and metatranscriptomic samples was done using HUMAnN2 v0.11.1 [48] and MetaPhlAn2 v2.7.7 [49]. To assess the contribution of various SFT pathway encoding organisms to total transcription of the SFT pathway, we mapped all of the trimmed reads from the 1,090 metatranscriptomes to all of the SFT pathway-encoding genomes we identified in our NCBI search (total of 83) with bbmap (v37.61, sourceforge.net/projects/bbmap/) using a minid of 97%. Additionally, we used the same pipeline and settings to map the trimmed reads from the 1,090 metatranscriptomes to *hpsGH*, *islAB*, and *dsrABC* genes identified in genomes of 16 representative *dsrAB*-containing human gut bacteria (see previous section). Feature counting was done using featureCounts within the Subread package (v1.6.3) [35]; reads that mapped equally well to multiple locations were assigned as fractions of the uniquely mapping reads for the same region (-M --fraction). Read counts were then converted to reads per kilobase per million reads (RPKM) or reads per kilobase (RPK). To estimate

the relative abundance of the SFT pathway in the 1,090 metatranscriptomes, we analyzed all of the metatranscriptome samples using HUMANn2 with default settings and then manually added the RPK values from each organism found to be expressing the SFT pathway as well as the sum pathway RPK values to the pathway abundance output from HUMANn2 prior to normalizing all of the RPK values to relative abundance using the humann2_renorm_table script provided in HUMAnN2. Proportional contribution of each organism to total expression of the SFT pathway was calculated as a ratio between the RPKM value for the organism of interest divided by the total RPKM value for the pathway in that sample. We compared mean expression (normalized RPKM values) of the SFT pathway and the DsrAB-DsrC pathway between the different cohorts in the metatranscriptomes (non-IBD vs UC, non-IBD vs CD, and UC vs CD) using a one-factor ANOVA test followed by Tukey's test; there were no statistically discernible differences in the expression means of both pathways between the different cohorts.

***E. rectale* transcription network analysis.** Downloaded and trimmed reads (processed as described above) from the 1,090 gut metatranscriptomes were mapped to the *E. rectale* ATCC 33656 genome using bbmap (v37.61, sourceforge.net/projects/bbmap/) with at a minid of 97%. Feature counting was done using featureCounts within the Subread package (v1.6.3) [35]. Samples with greater than ⅝ of all *E. rectale* ATCC 33656 genes with at least 1 read mapped (n = 244) were selected for network construction. Raw read counts for these 244 samples were then converted to copies per million counts (cpm) and normalized using a trimmed mean of M-values (TMM) between each pair of samples using calcNormFactors (edgeR, v3.9) [36]. The normalized matrix was then used as input into SPIEC-EASI (v1.0.5) [50] to construct a sparse and compositionally robust gene expression network using the following settings: method='mb', lambda.min.ratio=0.1, nlambda=10, rep.num=60. Combinations of different lambda min ratios ($1e^{-3}$ to $1e^{-1}$) and nlambda (10 to 100) were used to find a stability threshold near the target (0.05); a lambda min ratio of 0.1 and nlambda of 10 yielded an optimal stability threshold of 0.047. Significant correlations between gene pairs (positive or negative interaction strength of >0.099) with any functional prediction (n=1,349 unique genes, 2,326 gene-pairs) were then imported into Cytoscape (v3.7.1) [51] to visualize the network. Significant clusters were identified using ClusterONE [52] with the following settings: min. size=4, min.density=auto, edge.weights=unweighted. The list of significantly upregulated *E. rectale* ATCC 33656 genes from the SQ-amended fecal microcosms (see metatranscriptomics above) was then cross referenced with this network to identify co-expressed gene clusters that respond to SQ amendment.

Phylogenetic and phylogenomic tree construction. Phylogenomic analysis of the 93 MAGs from the fecal incubations was performed with GToTree (v1.4.2) [53] using 74 bacterial single-copy genes and a minimum 70% hit fraction for a MAG to be included in

the analysis. With this restriction, 66 MAGs were retained for further analysis. FastTree2 [41] was then used to construct approximate maximum likelihood trees using the JTT+CAT model. Comparative phylogenetic analysis of the glycoside hydrolase family 31 (which includes YihQ/SftG sulfoquinovosidases) was done by downloading the glycoside hydrolase family 31 seed alignment from the pfam database (780 amino acid sequences), aligning the sequences using MAFFT (v7.427) [39] (FFT-NS-i-x2 strategy), and then adding 219 putative novel YihQ sequences (70 characterized glycoside hydrolase family 31 proteins, 70 amino acid hits from the Uniprot database using the YihQ/SftG HMM at an e-value of $1e^{-6}$, and 79 unique YihQ/SftG homologs from the 83 genomes encoding the SFT pathway) and an additional 8,696 glycoside hydrolase 31 sequences using the --add and --keeplength options. The 8,696 glycoside hydrolase family 31 sequences correspond to 9,126 amino acid sequences from uniprot reference proteomes that matched the glycoside hydrolase HMM with an e-value of $1e^{-40}$ or less and then clustered at 90% identity using usearch. TrimAl (v1.4.rev15) was used in strict mode to trim the alignment to a final length of 284 amino acid positions. An approximate maximum likelihood tree was then constructed from the alignment using the JTT+CAT model. For comparative phylogenetic analysis of pyruvate formate-lyase like enzymes and to distinguish isethionate sulfite-lyases (IslA) from DHPS sulfite-lyases (HpsG), we retrieved amino acid sequences of known choline trimethylamine-lyases, 4-hydroxyphenylacetate decarboxylases, hydroxyproline dehydratases, aryl- alkyl-succinate synthases, glycerol dehydratases, and pyruvate formate-lyases from the KEGG ligand enzyme nomenclature database (abbreviation E.C.) and clustered them at 90% identity using usearch (v11.0.667) [40]. All of the sequences were aligned with MAFFT (v7.427) [39] using the FFT-NS-i-x2 strategy. 23 glycyl radical enzyme sequences, including known isethionate sulfite-lyases, from genomes of members of the family *Desulfovibrionaceae* originating from the human gut were added to the 1,204 reference amino acid sequence alignment with MAFFT using the --add and --keeplength options. TrimAl (v1.4.rev15) [54] was used in strict mode to trim the alignment to a final length of 569 positions. An approximate maximum likelihood tree was then constructed from the alignment with FastTree2 using the JTT+CAT model.

B. Supplementary Figures S1-S7

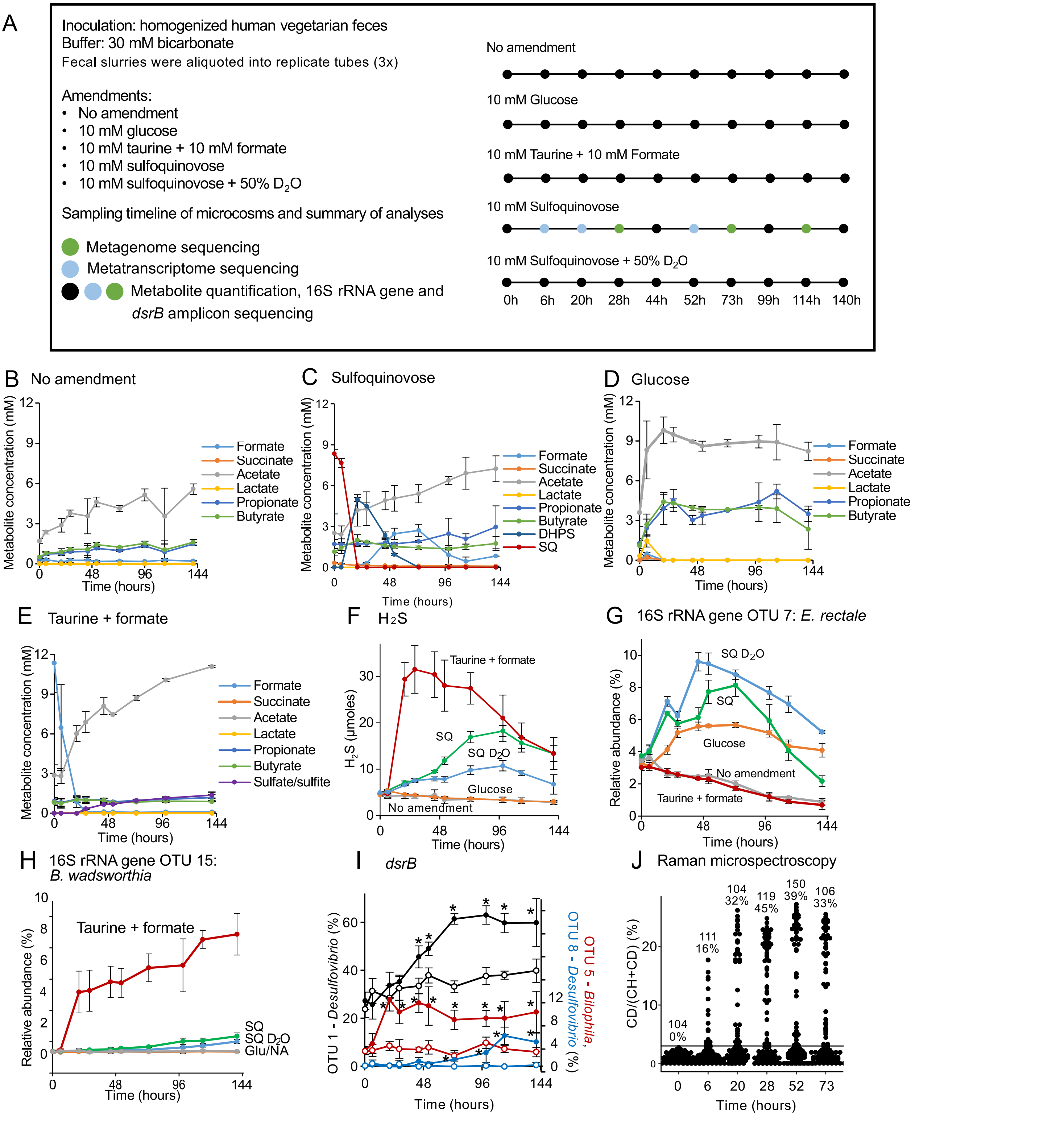

Fig. S1 Sulfoquinovose is rapidly degraded in anoxic microcosms with human feces. a Overview of fecal microcosm experimental plan. Five microcosm treatments were constructed in triplicate and subsampled at 10 time points. At all time points, metabolite quantification and 16S rRNA gene and *dsrB* amplicon sequencing were performed. Time points selected for metagenome sequencing are shown as green dots. Time points selected for metatranscriptome sequencing are shown as blue dots. **b-f** Microbial metabolite dynamics in microcosms **b**) without amendment, **c**) with 10 mM SQ, **d**) with 10 mM glucose, **e**) with 10 mM taurine plus 10 mM formate. In **b**, residual microbial activity showed no endogenously produced DHPS or H_2S. In **c**, added SQ is rapidly degraded to DHPS and acetate, while internally produced formate is consumed likely as an electron donor for sulfite respiration. In **c**, glucose addition promotes microbial production of mainly acetate, propionate, and butyrate. In **e**, formate is rapidly consumed as an electron donor for taurine-derived sulfite respiration with the production of more acetate. Sulfite accumulation after the depletion of formate might be due to lack of sufficient electron donor. An accumulation of hydroxyacetone as for the *B. wadsworthia* pure culture (Fig. S5a) was not detectable in the microcosms, suggesting consumption of hydroxyacetone by other microorganisms. In **f**, comparison of H_2S amounts in the five microcosm treatments indicated strong production of H_2S in the presence of taurine (serving as a positive control for organosulfonate respiration) and of SQ (both SQ alone and in the presence of 50% D_2O). (**g**, **h**) Relative abundance of *E. rectale* (OTU 7, panel **g**) and *B. wadsworthia* (OTU 15, panel **h**) 16S rRNA genes over time in the 5 microcosm treatments. **i** Three *dsrB* OTUs showed significant increases over time in SQ-amended microcosms (closed circles) compared to no amendment controls (open circles). *dsrB* OTUs were characterized at the genus level as *Desulfovibrio* (OTU 1, black lines; OTU 8, blue lines) and *Bilophila* (OTU 5, red lines). * indicate time points with significant differences in relative abundances between the no amendment and SQ-amended microcosms ($P < .01$). j Quantification of %CD-labeling using Raman microspectroscopys of individual microbial cells in SQ-amended microcosms containing 50% heavy water (D_2O). The total number of cells measured at each time point is shown with the percent of those cells with a %CD above a threshold cutoff of 3.54 %CD (black bar). In panels b-i, points represent averages of triplicate measures. Error bars represent one standard deviation. SQ, sulfoquinovose; DHPS, 2,3-dihydroxypropane-1-sulfonate; OTU, operational taxonomic unit; Glu, glucose treatment; NA, no amendment control.

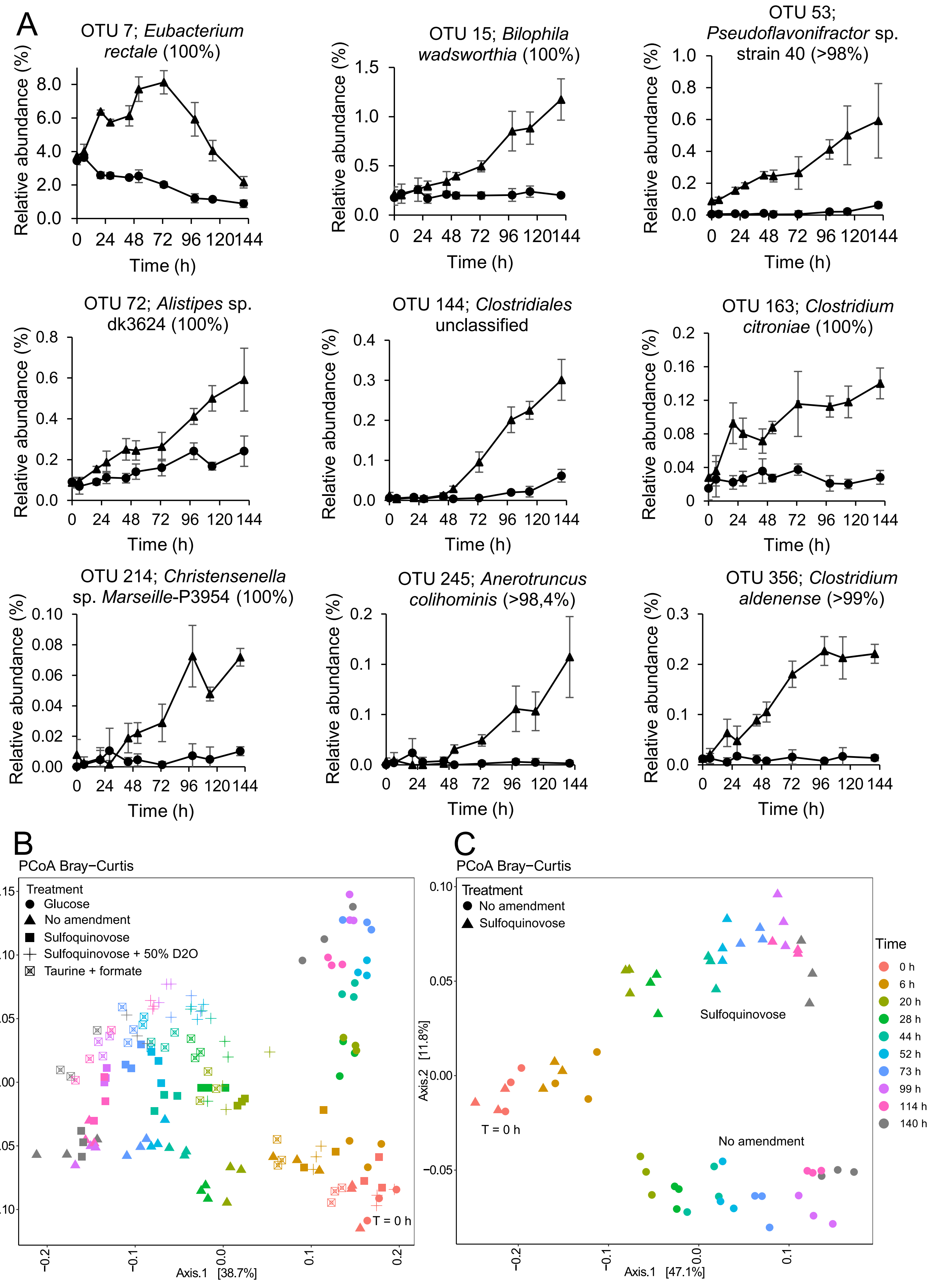

Fig. S2 Addition of sulfoquinovose to human feces microcosms triggers only minor shifts in microbiota composition. a Relative abundance of the nine OTUs (out of 791 OTUs total) that showed significant increases ($P < .01$) in SQ-amended microcosms (triangles) compared to no-amendment controls (circles). Points represent averages of triplicate measures. Error bars represent one standard deviation. Identity of the OTUs was revealed by phylogenetic placement into a reference tree with close relatives. Percentages in brackets indicate the 16S rRNA gene similarity to the closest reference species. (**b**, **c**) Principal component analysis plotting the shifts in microcosm beta-diversity (Bray-Curtis dissimilarity) over time. Points represent 16S rRNA gene amplicon libraries generated from biologically replicated microcosms. In **b**, all treatments are shown. In **c**, only the no-amendment and sulfoquinovose treatments are shown to emphasize community shifts driven by SQ. SQ, sulfoquinovose; OTU, operational taxonomic unit.

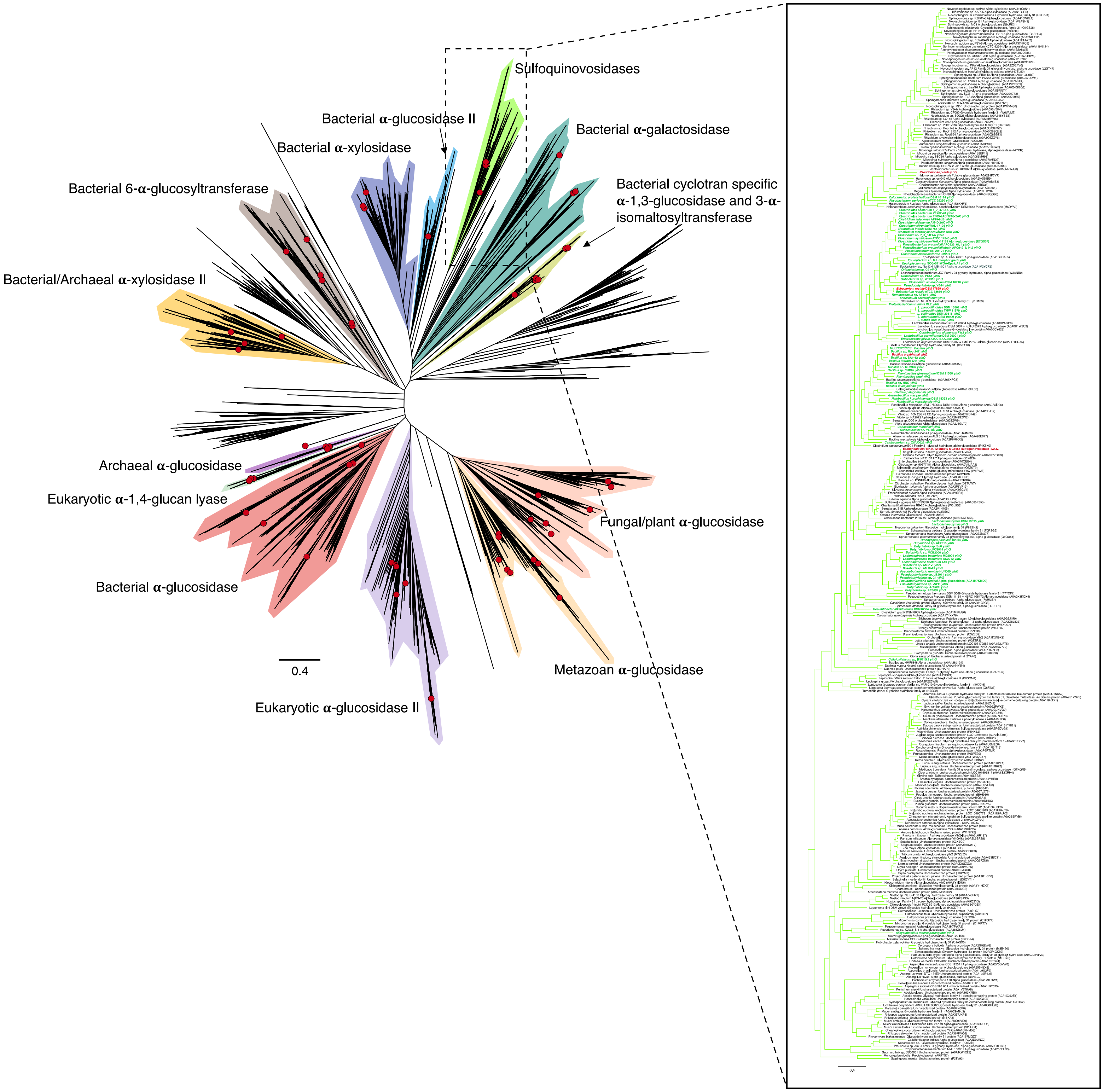

Fig. S3 Comparative phylogenetic analysis of glycoside hydrolase family 31 enzymes, including YihQ/SftG sulfoquinovosidases. The glycoside hydrolase family 31 phylogeny was reconstructed using FastTree2 [41] and the scale bar indicates the number of amino acid substitutions per position. The boxed inset depicts the phylogeny of putative and biochemically characterized (depicted in red) YihQ/SftG sulfoquinovosidases. Organisms that were identified to encode all four core enzymes of the SFT fermentation pathway are highlighted in green. Experimental characterizations of function from the CAZy database are depicted as red circles on the branch tips.

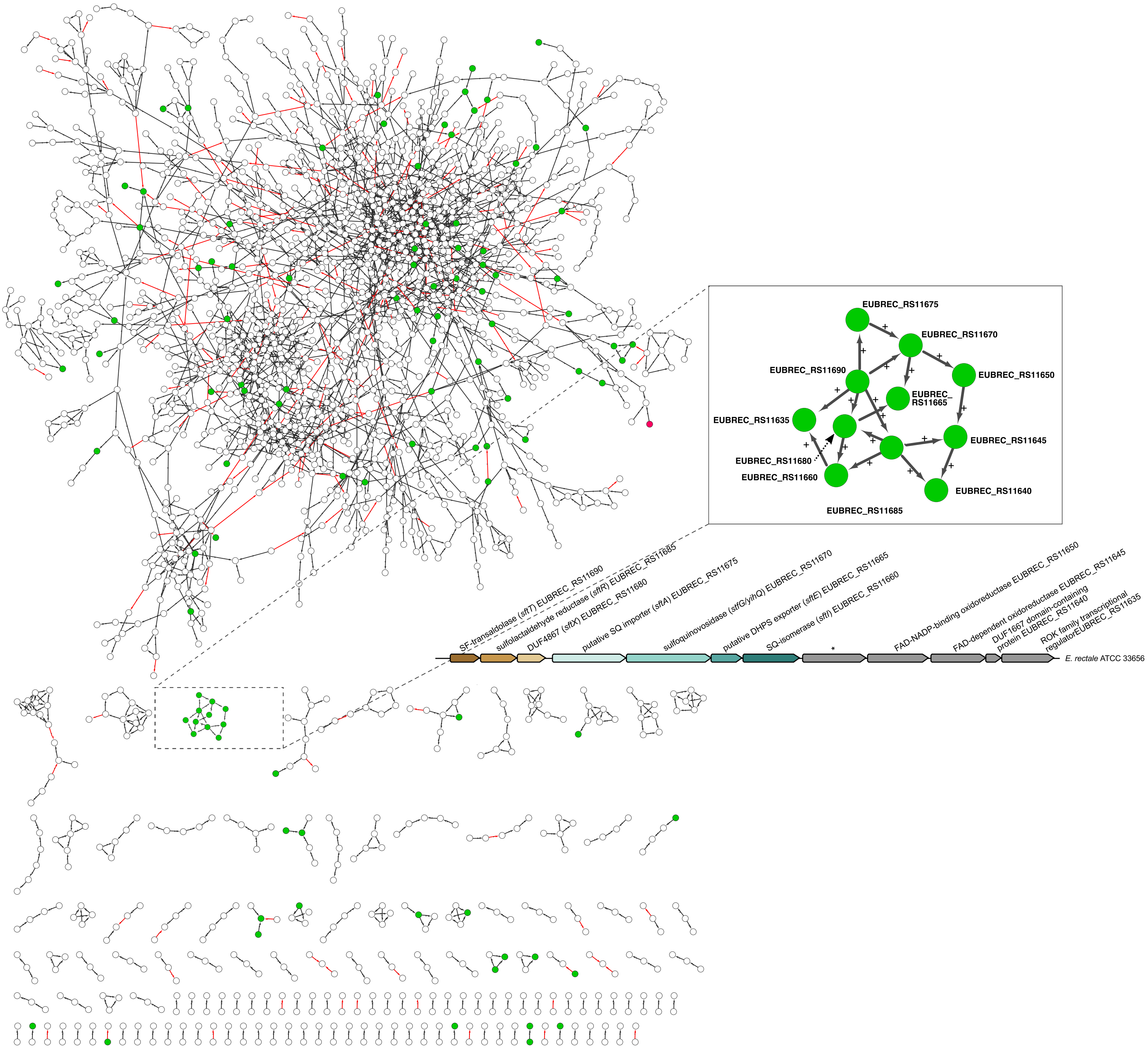

Fig. S4 A genome-wide *E. rectale* transcription network reveals co-expression of a sulfoquinovosidase gene-containing gene cluster in human fecal samples. Expression of yihQ/sftG and 10 co-localized genes from *E. rectale* ATCC 33656 are positively correlated to one another across 1,090 human gut metatranscriptomes [46, 47, 55]. Circles (nodes) represent individual E. rectale genes and arrows depict significant interactions (edges); grey arrows represent significant positive (> 0.099) correlations and red arrows represent significant (< -0.099) negative correlations between genes. Nodes highlighted in green are genes whose expressions are significantly upregulated in the presence of 10 mM SQ in human fecal microcosms. Clusters are arranged in order of cluster size, with the largest cluster at the top of the network. The only cluster composed solely of genes upregulated in SQ-amended fecal microcosms is the SFT fermentation gene cassette. The inset depicts the identities of the individual genes in this cluster, with the operon from the genome sequence below the inset. The gene denoted with an asterisk (EUBREC_RS11655) encodes a putative transcriptional regulator and does not appear in the network due to low interaction strength with any node (correlation 0.000 < * < 0.099).

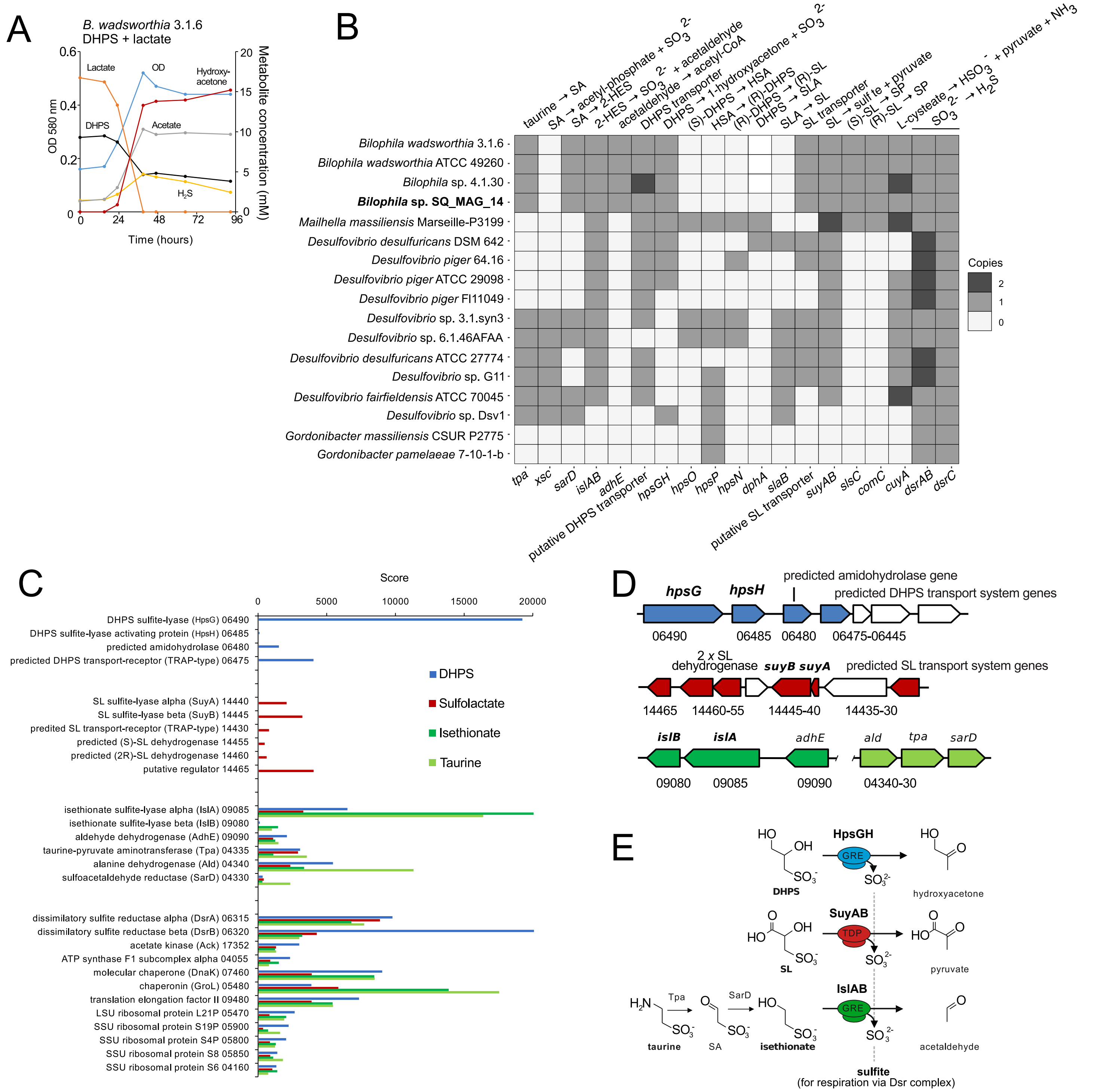

Fig. S5 Identification of a DHPS-desulfonating enzyme (HpsG) in *B. wadsworthia* 3.1.6 and its distribution among selected sulfur compound-metabolising human gut bacteria. **a** Growth of *B. wadsworthia* 3.1.6 (OD580 nm; blue line) with DHPS as an electron acceptor (black line) and lactate as an electron donor (orange line), showing production of acetate (gray line), H_2S (yellow line), and of hydroxyacetone (red line). Production of hydroxyacetone from the desulfonation of DHPS implied a reaction (attributed to HpsG) analogous to the desulfonation of isethionate [4]. **b** Presence/absence of organosulfur metabolism genes in the genomes of selected *dsrABC*-encoding microbes from the human gut and the Bilophila sp. MAG from the microcosm (depicted in boldface). **c** Differential proteomics of *B. wadsworthia* 3.1.6 grown with lactate and either DHPS, SL, isethionate or taurine as electron acceptors (each n=1) illustrates substrate-specific induction of different (predicted) pathway enzymes. For all identified inducible genes, RefSeq locus tag numbers are shown (prefix HMPREF0179_RS) (panels **c**, **d**). In addition, HpsGH in *Desulfovibrio desulfurican*s strain DSM642 (panel **b**) was also strongly induced during DHPS utilization, as confirmed by differential proteomics in comparison to sulfate, and strain DSM642 also excreted hydroxyacetone during growth with DHPS (data not shown). (**d**) Overview of the identified genes located in separate gene clusters in *B. wadsworthia* 3.1.6 that are strongly induced during growth with either DHPS (blue), SL (red), or isethionate (dark green), and with taurine (light green, for taurine metabolism via the isethionate-desulfonation pathway [4]). In addition to the inducible TRAP-transport-system receptors identified by proteomics (**c**), the gene clusters for DHPS and SL also encode TRAP-transport system membrane permeases that were not identified by total proteomics (indicated in white); they most likely mediate transport of DHPS and SL, respectively. The two SL dehydrogenase genes identified in the SL-utilization gene cluster (14460, 14455 in **c**, **d**) are homologous to SlcC and ComC of Chromohalobacter salexigens, which catalyze an interconversion of (S)-3-sulfolactate to (R)-3-sulfolactate via sulfopyruvate, respectively [56] (not shown in **d**, shown in **e**). **e** Illustration of the enzyme reaction for the DHPS-inducible GRE (blue), shown desulfonating DHPS to hydroxyacetone and sulfite (DHPS sulfite-lyase, HpsGH) in a reaction analogous to that of the first identified desulfonating GRE, isethionate sulfite-lyase (green) (IslAB) [4], and the desulfonation reaction of 3-sulfolactate sulfo-lyase (SuyAB) (red), a thiamindiphosphat [TDP] dependent enzyme [5]. The desulfonation reaction of DHPS to sulfite and hydroxyacetone was detected in strictly anoxic cell-free extracts of DHPS-grown cells (Fig. 2c) but not in the presence of oxygen (Fig. 2c). Note that isethionate sulfite-lyase IslAB of B. wadsworthia 3.1.6 cannot catalyze a desulfonation reaction with DHPS [4]. 2-HES, 2-hydroxyethane-1-sulfonic acid (isethionate); DHPS, 2,3-dihydroxypropane-1-sulfonate; HSA, 2-oxo-3-hydroxy-propane-1-sulfonate; SLA, 3-sulfolactaldehyde; SL, 3-sulfolactate; SA, sulfoacetaldehyde; SP, sulfopyruvate; GRE, glycyl radical enzyme.

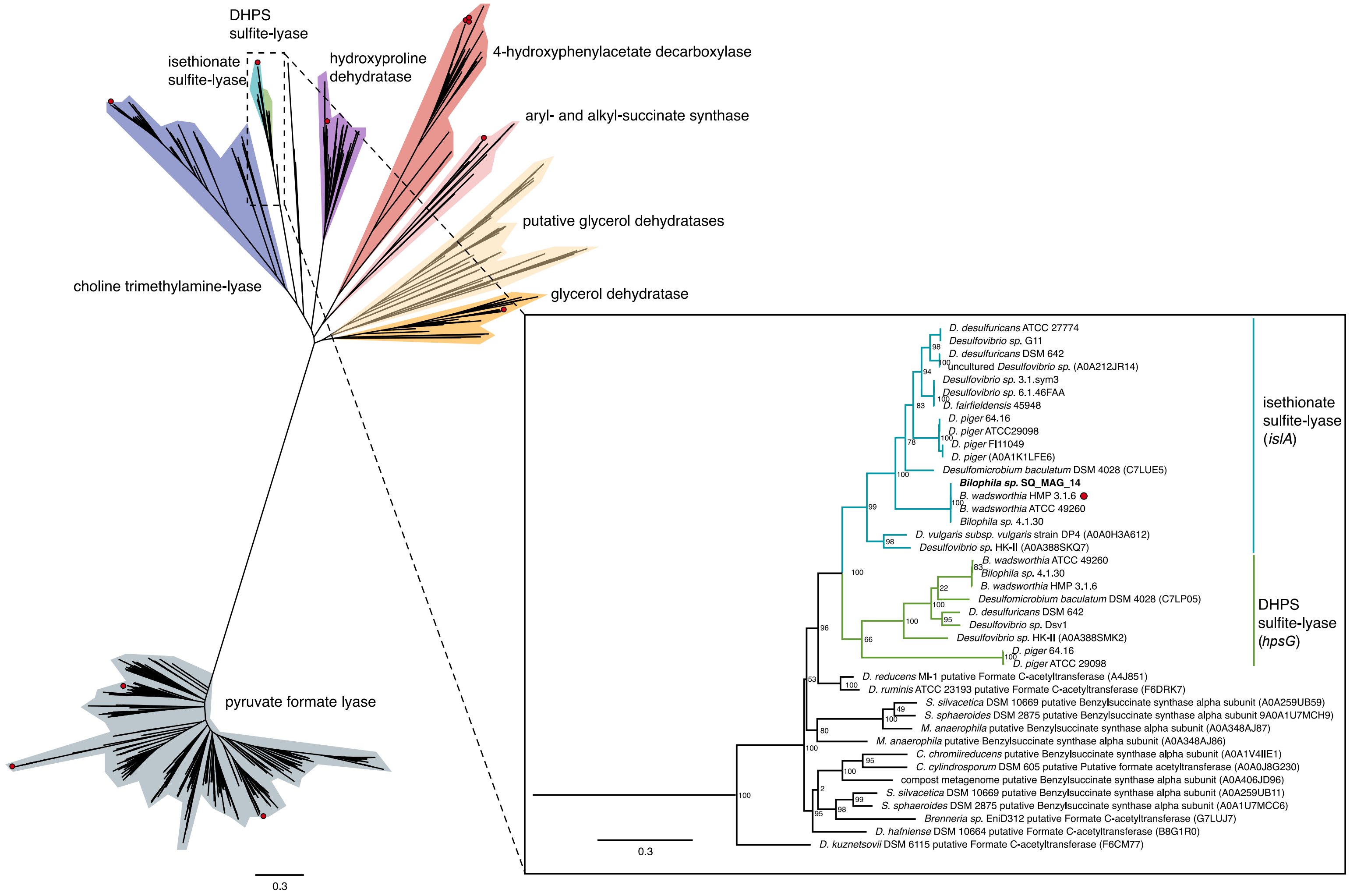

Fig. S6 Genes for the DHPS sulfite-lyase (*hpsG*) and isethionate sulfite-lyase (*islA*) of *B. wadsworthia* are paralogous. Phylogenetic analysis of pyruvate formate lyases and glycyl radical enzymes from the KEGG ligand enzyme nomenclature database and *Desulfovibrionaceae* family genomes shows that isethionate sulfite-lyases (encoded by *islA*) and DHPS sulfite-lyases (encoded by *hpsG*) are closely related but phylogenetically distinct. Amino acid identities between IslA and HpsG ranged from 54% to 64%; and were greater than 76% and 81% within HpsG and IslA groups, respectively. Experimental characterization of function from the Uniprot database are depicted as red circles on the branch tips. DHPS, 2,3-dihydroxypropane-1-sulfonate.

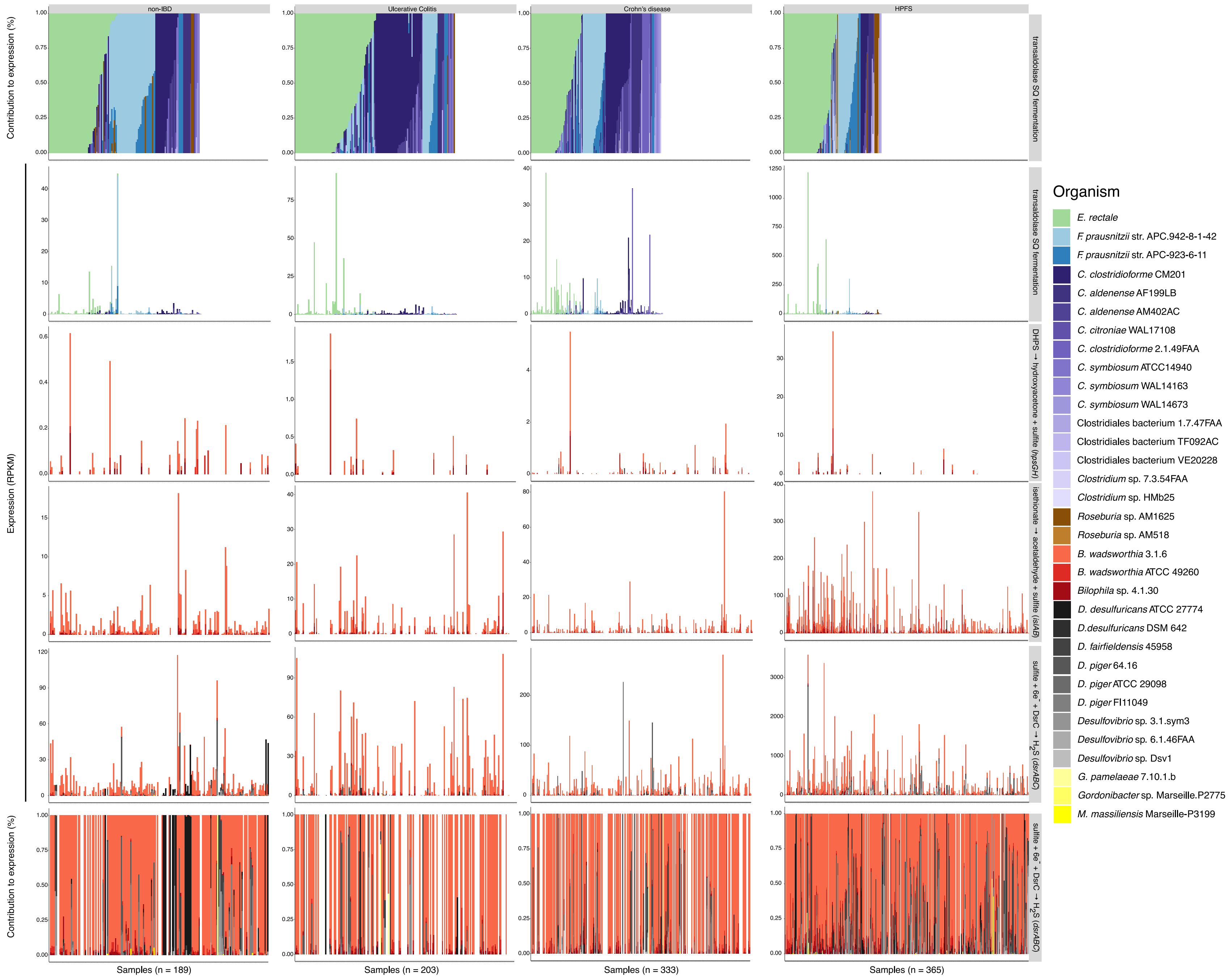

Fig. S7 *Eubacterium rectale* and *Bilophila wadsworthia* are the dominant contributors to expression of the SFT fermentation and H_2S production pathways in human stool metatranscriptomes, respectively. Percentage and normalized read contribution (in RPKM) of various Firmicutes and gut sulfidogens to expression of the SFT pathway, *hpsG*-dependent DHPS catabolism (*hpsGH*), taurine/isethionate catabolism (*islAB*), and sulfite reduction to sulfide (*dsrABC*) in publicly available human stool metatranscriptomes from a non-IBD cohort (n=130), ulcerative colitis cohort (n=203), Crohn's disease cohort (n=333), and HPFS study (n=365) [46, 47, 55, 57]. Each bar corresponds to 1 sample. The samples are aligned between the panels and ordered according to SFT pathway expression. Contributions to expression (%) are fractional with 1.0 corresponding to 100%. Abbreviations: IBD, inflammatory bowel disease; HPFS, health professionals follow-up study; RPKM, reads per kilobase per million reads; SQ, sulfoquinovose; DHPS, 2,3-dihydroxypropane-1-sulfonate.

C. Supplementary Tables S1-S12 Legends

Table S1. 16S rRNA-targeted oligonucleotide probes used for fluorescence *in situ* hybridization. Probes and their respective unlabeled competitors were used in equimolar concentrations during hybridization. (a) According to Alm *et al*. [58]. (b) Formamide concentration in the hybridization buffer. (c) SILVA test probe match was performed with SSU Ref132 database containing 2,090,668 bacterial and archaeal 16S rRNA sequences [10]. Coverage is the percentage of sequences within the SILVA target taxon with a full match to the probe sequence. The number of non-target hits indicates the total number of sequences outside the respective SILVA target taxon with a full match to the probe sequence.

Table S2. SFT pathway-encoding bacterial genomes in the NCBI database.

Table S3. SFT pathway-encoding genomes/MAGs from a MAG/genome database of human gut microorganisms, The MAG/genome database is available at http://segatalab.cibio.unitn.it/data/Pasolli_et_al.html and http://opendata.lifebit.ai/table/?project=SGB.

Table S4. 16S rRNA gene amplicon OTU table. Summary of 16S rRNA gene amplicon read counts for each microcosm treatment (No amendment, 10 mM Glucose, 10 mM taurine + 10 mM formate, 10 mM sulfoquinovose, and 10 mM sulfoquinovose + 50% D_2O), replicates, and time points. OTUs were clustered at 97% and taxonomic assignment is shown (domain_id, phylum_id, class_id, order_id, family_id, and genus_id) with the associated confidence scores (domain_conf, phylum_conf, class_conf, order_conf, family_conf, and genus_conf). Contaminant OTUs were determined from replicate DNA extraction blank controls and no-template PCR controls. Removed contaminant OTUs (149 OTUs) are presented at the bottom of this sheet.

Table S5. Results of 16S rRNA gene DESeq2 analyses. Results from significance testing using DESeq2 comparing OTU counts between 16S rRNA gene libraries generated from fecal microcosms receiving no amendment vs. 10 mM sulfoquinovose across paired time points. Only OTUs and timepoints having a significantly greater relative abundance in the sulfoquinovose-amended microcosms compared with the no amendment control are shown. OTU_name; Time_point_(hours), time point of incubation in which DNA was harvested from; default output from DESeq2 (baseMean; log2FoldChange; lfcSE, log2FoldChange standard error; stat; pvalue; padj, adjusted pvalue); followed by taxonomic assignment (domain_id, phylum_id, class_id, order_id, family_id, and genus_id) and confidence scores(domain_conf, phylum_conf, class_conf, order_conf, family_conf, and genus_conf) [14].

Table S6. *dsrB* amplicon OTU table. Summary of *dsrB* amplicon read counts for each microcosm treatment (No amendment, 10 mM Glucose, 10 mM taurine + 10 mM formate, 10 mM sulfoquinovose, and 10 mM sulfoquinovose + 50% D_2O), replicates, and time points. OTUs were clustered at 97% and taxonomic assignment is shown (Gene_type, Domain, Phylum, Class, Order, Family, and Genus) according to Pelikan et al., 2015 and

Müller et al. 2015 [15, 20]. No contaminant OTUs were detected as determined from replicate DNA extraction blank controls and no-template PCR controls.

Table S7. Results of *dsrB* DESeq2 analyses. Results from significance testing using DESeq2 comparing OTU counts between *dsrB* libraries generated from fecal microcosms receiving no amendment vs. 10 mM sulfoquinovose across paired time points. Only OTUs and timepoints having a significantly greater relative abundance in the sulfoquinovose-amended microcosms compared with the no amendment control are shown. OTU_name; Time_point_(hours), time point of incubation in which DNA was harvested from; default output from DESeq2 (baseMean; log2FoldChange; lfcSE, log2FoldChange standard error; stat; pvalue; padj, adjusted pvalue); followed by taxonomic assignment (Gene_type, Domain, Phylum, Class, Family, Genus) [15, 20].

Table S8. Metagenome assembled genomes from microcosms. Summary of metagenome assembled genomes (MAGs) dereplicated across three metagenomic datasets prepared from SQ-amended microcosms (time points 28, 73, and 144 h). MAG ID (column 1) sorted by MAG completeness. Bolded entries indicate MAGs identified as *Eubacterium rectale* and *Bilophila wadsworthia* according to average nucleotide identities (ANI) with genomes of publicly available type strains. Genome taxonomy based on concatenated genes sets (column 2) according to the Genome Taxonomy Database [28]. MAG quality was assessed using CheckM [26] with columns 3-13 showing resultant summary data. Strain heterogeneity (%, column 14) was determined using dRep [27]. Presence of 16S SSU rRNA sequences in each MAG (column 15) was determined with nhmmer [29] using rfam [30] models for bacterial and archaeal SSU rRNAs (RFAM: RF00177, RF01959). Minimum overlap between sequence and model was 300 nucleotides. Sequences were classified using the RDPclassifer [17] as implemented in Mothur. The number of tRNAs identified and the number of amino acids encoded by identified tRNAs (columns 16 and 17, respectively) were determined using tRNAscan-SE [31].

Table S9. *E. rectale* genome average nucleotide identity (ANI) matrix.

Table S10. *B. wadsworthia* genome ANI matrix.

Table S11. Significance values for *E. rectale* transcripts expressed in microcosms. Locus tags, gene annotations, normalized read counts, and EdgeR exact test results for read mapping to *Eubacterium rectale* ATCC 33656 in the triplicate SQ-amended microcosms at 6 hours, 20 hours, and 52 hours. P-values highlighted in red denote significantly differentially expressed genes ($P < 0.05$) at 20 hours compared to 6 hours. Genes highlighted in red denote genes that make up the SFT pathway gene cassette.

Table S12. Significance values for *B. wadsworthia* transcripts expressed in microcosms. Locus tags, gene annotations, normalized read counts, and EdgeR exact test results for read mapping to *Bilophila wadsworthia* 3.1.6 in the SQ-amended microcosms at 6 hours, 20 hours, and 52 hours. P-values highlighted in red denote significantly differentially expressed genes ($P < 0.05$) at 20 hours compared to 6 hours.

D. Supplementary Files

File S1 YihQ-SftG.hmm. YihQ/SftG-specific HMM constructed using signature residues reported by Speciale *et al*. [38].

E. Supplementary References

1. Kuriyama I, Musumi K, Yonezawa Y, Takemura M, Maeda N, Iijima H, et al. Inhibitory effects of glycolipids fraction from spinach on mammalian DNA polymerase activity and human cancer cell proliferation. *J Nutr Biochem* 2005; **16**: 594–601.
2. Daims H, Stoecker K, Wagner M. Fluorescence *in situ* hybridization for the detection of prokaryotes. In: Osborn A, Smith C (eds). *Advanced Methods in Molecular Microbial Ecology*. 2005. Bios-Garland, Abingdon, UK, pp 213–239.
3. Berry D, Schwab C, Milinovich G, Reichert J, Ben Mahfoudh K, Decker T, et al. Phylotype-level 16S rRNA analysis reveals new bacterial indicators of health state in acute murine colitis. *ISME J* 2012; **6**: 2091–2106.
4. Peck SC, Denger K, Burrichter A, Irwin SM, Balskus EP, Schleheck D. A glycyl radical enzyme enables hydrogen sulfide production by the human intestinal bacterium. *Proc Natl Acad Sci U S A* 2019; **116**: 3171–3176.
5. Burrichter A, Denger K, Franchini P, Huhn T, Müller N, Spiteller D, et al. Anaerobic Degradation of the Plant Sugar Sulfoquinovose Concomitant With H_2S Production: *Escherichia coli* K-12 and *Desulfovibrio* sp. Strain DF1 as Co-culture Model. *Front Microbiol* 2018; **9**: 2792.
6. Frommeyer B, Fiedler AW, Oehler SR, Hanson BT, Loy A, Franchini P, et al. Environmental and intestinal phylum *Firmicutes* bacteria metabolize the plant sugar sulfoquinovose via a 6-deoxy-6-sulfofructose transaldolase pathway. *iScience* 2020; 101510.
7. Cline JD. Spectrophotometric determination of hydrogen sulfide in natural waters. *Limnol Oceanogr* 1969; **14**: 454–458.
8. Denger K, Weiss M, Felux A-K, Schneider A, Mayer C, Spiteller D, et al. Sulphoglycolysis in *Escherichia coli* K-12 closes a gap in the biogeochemical sulphur cycle. *Nature* 2014; **507**: 114–117.
9. Ludwig W, Strunk O, Westram R, Richter L, Meier H, Yadhukumar, et al. ARB: a software environment for sequence data. *Nucleic Acids Res* 2004; **32**: 1363–1371.
10. Quast C, Pruesse E, Yilmaz P, Gerken J, Schweer T, Yarza P, et al. The SILVA ribosomal RNA gene database project: improved data processing and web-based tools. *Nucleic Acids Res* 2013; **41**: D590–6.
11. Berry D, Mader E, Lee TK, Woebken D, Wang Y, Zhu D, et al. Tracking heavy water (D_2O) incorporation for identifying and sorting active microbial cells. *Proc Natl Acad Sci USA* 2015; **112**: E194–203.
12. Daims H, Brühl A, Amann R, Schleifer KH, Wagner M. The domain-specific probe EUB338 is insufficient for the detection of all Bacteria: development and evaluation of a more comprehensive probe set. *Syst Appl Microbiol* 1999; **22**: 434–444.
13. Griffiths RI, Whiteley AS, O'Donnell AG, Bailey MJ. Rapid method for coextraction of DNA and RNA from natural environments for analysis of ribosomal DNA- and rRNA-based microbial community composition. *Appl Environ Microbiol* 2000; **66**: 5488–5491.
14. Herbold CW, Pelikan C, Kuzyk O, Hausmann B, Angel R, Berry D, et al. A flexible and economical barcoding approach for highly multiplexed amplicon sequencing of diverse target genes. *Front Microbiol* 2015; **6**: 731.
15. Pelikan C, Herbold CW, Hausmann B, Müller AL, Pester M, Loy A. Diversity analysis of

sulfite- and sulfate-reducing microorganisms by multiplex *dsrA* and *dsrB* amplicon sequencing using new primers and mock community-optimized bioinformatics. *Environ Microbiol* 2016; **18**: 2994–3009.

16. Edgar RC. UPARSE: highly accurate OTU sequences from microbial amplicon reads. *Nat Methods* 2013; **10**: 996–998.
17. Wang Q, Garrity GM, Tiedje JM, Cole JR. Naive Bayesian classifier for rapid assignment of rRNA sequences into the new bacterial taxonomy. *Appl Environ Microbiol* 2007; **73**: 5261–5267.
18. Schloss PD, Westcott SL, Ryabin T, Hall JR, Hartmann M, Hollister EB, et al. Introducing mothur: open-source, platform-independent, community-supported software for describing and comparing microbial communities. *Appl Environ Microbiol* 2009; **75**: 7537–7541.
19. Kumar S, Stecher G, Tamura K. MEGA7: Molecular Evolutionary Genetics Analysis Version 7.0 for Bigger Datasets. *Mol Biol Evol* 2016; **33**: 1870–1874.
20. Müller AL, Kjeldsen KU, Rattei T, Pester M, Loy A. Phylogenetic and environmental diversity of DsrAB-type dissimilatory (bi)sulfite reductases. *ISME J* 2015; **9**: 1152–1165.
21. McMurdie PJ, Holmes S. phyloseq: an R package for reproducible interactive analysis and graphics of microbiome census data. *PLoS One* 2013; **8**: e61217.
22. Love MI, Huber W, Anders S. Moderated estimation of fold change and dispersion for RNA-seq data with DESeq2. *Genome Biol* 2014; **15**: 550.
23. Nikolenko SI, Korobeynikov AI, Alekseyev MA. BayesHammer: Bayesian clustering for error correction in single-cell sequencing. *BMC Genomics* 2013; **14 Suppl 1**: S7.
24. Nurk S, Meleshko D, Korobeynikov A, Pevzner PA. metaSPAdes: a new versatile metagenomic assembler. *Genome Res* 2017; **27**: 824–834.
25. Kang DD, Froula J, Egan R, Wang Z. MetaBAT, an efficient tool for accurately reconstructing single genomes from complex microbial communities. *PeerJ* 2015; **3**: e1165.
26. Parks DH, Imelfort M, Skennerton CT, Hugenholtz P, Tyson GW. CheckM: assessing the quality of microbial genomes recovered from isolates, single cells, and metagenomes. *Genome Res* 2015; **25**: 1043–1055.
27. Olm MR, Brown CT, Brooks B, Banfield JF. dRep: a tool for fast and accurate genomic comparisons that enables improved genome recovery from metagenomes through de-replication. *ISME J* 2017; **11**: 2864–2868.
28. Parks DH, Chuvochina M, Waite DW, Rinke C, Skarshewski A, Chaumeil P-A, et al. A standardized bacterial taxonomy based on genome phylogeny substantially revises the tree of life. *Nat Biotechnol* 2018; **36**: 996–1004.
29. Wheeler TJ, Eddy SR. nhmmer: DNA homology search with profile HMMs. *Bioinformatics* 2013; **29**: 2487–2489.
30. Kalvari I, Argasinska J, Quinones-Olvera N, Nawrocki EP, Rivas E, Eddy SR, et al. Rfam 13.0: shifting to a genome-centric resource for non-coding RNA families. *Nucleic Acids Res* 2018; **46**: D335–D342.
31. Lowe TM, Eddy SR. tRNAscan-SE: a program for improved detection of transfer RNA genes in genomic sequence. *Nucleic Acids Res* 1997; **25**: 955–964.
32. Jain C, Rodriguez-R LM, Phillippy AM, Konstantinidis KT, Aluru S. High throughput ANI analysis of 90K prokaryotic genomes reveals clear species boundaries. *Nat Commun* 2018; **9**: 5114.

33. Li H, Handsaker B, Wysoker A, Fennell T, Ruan J, Homer N, et al. The Sequence Alignment/Map format and SAMtools. *Bioinformatics* 2009; **25**: 2078–2079.
34. Bolger AM, Lohse M, Usadel B. Trimmomatic: a flexible trimmer for Illumina sequence data. *Bioinformatics* 2014; **30**: 2114–2120.
35. Liao Y, Smyth GK, Shi W. featureCounts: an efficient general purpose program for assigning sequence reads to genomic features. *Bioinformatics* 2014; **30**: 923–930.
36. Robinson MD, McCarthy DJ, Smyth GK. edgeR: a Bioconductor package for differential expression analysis of digital gene expression data. *Bioinformatics* 2010; **26**: 139–140.
37. Potter SC, Luciani A, Eddy SR, Park Y, Lopez R, Finn RD. HMMER web server: 2018 update. *Nucleic Acids Res* 2018; **46**: W200–W204.
38. Speciale G, Jin Y, Davies GJ, Williams SJ, Goddard-Borger ED. YihQ is a sulfoquinovosidase that cleaves sulfoquinovosyl diacylglyceride sulfolipids. *Nat Chem Biol* 2016; **12**: 215–217.
39. Katoh K, Standley DM. MAFFT multiple sequence alignment software version 7: improvements in performance and usability. *Mol Biol Evol* 2013; **30**: 772–780.
40. Edgar RC. Search and clustering orders of magnitude faster than BLAST. *Bioinformatics* 2010; **26**: 2460–2461.
41. Price MN, Dehal PS, Arkin AP. FastTree 2--approximately maximum-likelihood trees for large alignments. *PLoS One* 2010; **5**: e9490.
42. Pasolli E, Asnicar F, Manara S, Zolfo M, Karcher N, Armanini F, et al. Extensive Unexplored Human Microbiome Diversity Revealed by Over 150,000 Genomes from Metagenomes Spanning Age, Geography, and Lifestyle. *Cell* 2019; **176**: 649–662.e20.
43. Seemann T. Prokka: rapid prokaryotic genome annotation. *Bioinformatics* 2014; **30**: 2068–2069.
44. Hyatt D, Chen G-L, Locascio PF, Land ML, Larimer FW, Hauser LJ. Prodigal: prokaryotic gene recognition and translation initiation site identification. *BMC Bioinformatics* 2010; **11**: 119.
45. Browne HP, Forster SC, Anonye BO, Kumar N, Neville BA, Stares MD, et al. Culturing of 'unculturable' human microbiota reveals novel taxa and extensive sporulation. *Nature* 2016; **533**: 543–546.
46. Schirmer M, Franzosa EA, Lloyd-Price J, McIver LJ, Schwager R, Poon TW, et al. Dynamics of metatranscription in the inflammatory bowel disease gut microbiome. *Nat Microbiol* 2018; **3**: 337–346.
47. Abu-Ali GS, Mehta RS, Lloyd-Price J, Mallick H, Branck T, Ivey KL, et al. Metatranscriptome of human faecal microbial communities in a cohort of adult men. *Nat Microbiol* 2018; **3**: 356–366.
48. Franzosa EA, McIver LJ, Rahnavard G, Thompson LR, Schirmer M, Weingart G, et al. Species-level functional profiling of metagenomes and metatranscriptomes. *Nat Methods* 2018; **15**: 962–968.
49. Truong DT, Franzosa EA, Tickle TL, Scholz M, Weingart G, Pasolli E, et al. MetaPhlAn2 for enhanced metagenomic taxonomic profiling. *Nat Methods* 2015; **12**: 902–903.
50. Kurtz ZD, Müller CL, Miraldi ER, Littman DR, Blaser MJ, Bonneau RA. Sparse and compositionally robust inference of microbial ecological networks. *PLoS Comput Biol* 2015; **11**: e1004226.
51. Shannon P, Markiel A, Ozier O, Baliga NS, Wang JT, Ramage D, et al. Cytoscape: a software

environment for integrated models of biomolecular interaction networks. *Genome Res* 2003; **13**: 2498–2504.

52. Nepusz T, Yu H, Paccanaro A. Detecting overlapping protein complexes in protein-protein interaction networks. *Nat Methods* 2012; **9**: 471–472.
53. Lee MD. GToTree: a user-friendly workflow for phylogenomics. *Bioinformatics* 2019; **35**: 4162–4164.
54. Capella-Gutiérrez S, Silla-Martínez JM, Gabaldón T. trimAl: a tool for automated alignment trimming in large-scale phylogenetic analyses. *Bioinformatics* 2009; **25**: 1972–1973.
55. Mehta RS, Abu-Ali GS, Drew DA, Lloyd-Price J, Subramanian A, Lochhead P, et al. Stability of the human faecal microbiome in a cohort of adult men. *Nat Microbiol* 2018; **3**: 347–355.
56. Denger K, Cook AM. Racemase activity effected by two dehydrogenases in sulfolactate degradation by *Chromohalobacter salexigens*: purification of (S)-sulfolactate dehydrogenase. *Microbiology* 2010; **156**: 967–974.
57. Lloyd-Price J, Arze C, Ananthakrishnan AN, Schirmer M, Avila-Pacheco J, Poon TW, et al. Multi-omics of the gut microbial ecosystem in inflammatory bowel diseases. *Nature* 2019; **569**: 655–662.
58. Alm EW, Oerther DB, Larsen N, Stahl DA, Raskin L. The oligonucleotide probe database. *Appl Environ Microbiol* 1996; **62**: 3557–3559.
